# Maturation of Filopodia Shaft Adhesions Is Upregulated by Local Cycles of Lamellipodia Advancements and Retractions

**DOI:** 10.1371/journal.pone.0107097

**Published:** 2014-09-17

**Authors:** Wei Hu, Bernhard Wehrle-Haller, Viola Vogel

**Affiliations:** 1 Laboratory of Applied Mechanobiology, Department of Health Sciences and Technology, ETH Zurich, Zurich, Switzerland; 2 Department of Cellular Physiology and Metabolism, University Medical Center, University of Geneva, Geneva, Switzerland; King’s College London, United Kingdom

## Abstract

While cell-substrate adhesions that form between the protruding edge of a spreading cell and flat surfaces have been studied extensively, processes that regulate the maturation of filopodia adhesions are far less characterized. Since little is known about how the kinetics of formation or disassembly of filopodia adhesions is regulated upon integration into the lamellum, a kinetic analysis of the formation and disassembly of filopodia adhesions was conducted at the leading edge of β3-integrin-EGFP-expressing rat embryonic fibroblasts spreading on fibronectin-coated glass or on soft polyacrylamide gels. Filopodia β3-integrin adhesions matured only if the lamellipodium in their immediate vicinity showed cyclic protrusions and retractions. Filopodia β3-integrin shaft adhesions elongated rapidly when they were overrun by the advancing lamellipodium. Subsequently and once the lamellipodium stopped its advancement at the distal end of the filopodia β3-integrin adhesion, these β3-integrin shaft adhesions started to grow sidewise and colocalize with the newly assembled circumferential actin stress fibers. In contrast, the suppression of the cyclic protrusions and retractions of the lamellipodium by blocking myosin light chain kinase suppressed the growth of filopodia adhesion and resulted in the premature disassembly of filopodia adhesions. The same failure to stabilize those adhesions was found for the advancing lamellipodium that rapidly overran filopodia shaft adhesions without pausing as seen often during fast cell spreading. In turn, plating cells on soft polyacrylamide gels resulted in a reduction of lamellipodia activity, which was partially restored locally by the presence of filopodia adhesions. Thus filopodia adhesions could also mature and be integrated into the lamellum for fibroblasts on soft polyacrylamide substrates.

## Introduction

Cells use filopodia to explore the physical and biochemical characteristics of their environments [1]–[6], and the stabilization of filopodial contacts with the substrate (matrix) directs cell movement [2], [7], [8]. Filopodia thus play a central role in the recognition of (nano) structured surfaces [9], and support the migration of cells into nanofibrillar environments [8], [10], [11] thereby enabling angiogenesis [12] or cancer cell metastasis [13]. Filopodia spontaneously protrude from the edge of various cell types and are thus the first and farthest protruding cellular structures during cell spreading and migration [1]–[14]. They are composed of parallel filamentous actin bundles that are nucleated in the lamellipodia actin network via the proposed mechanisms of “de novo filament nucleation” or “convergence elongation” [15], [16], and get bundled by fascin [17], [18]. After cell seeding, filopodia were found to form the very first substrate contacts prior to cell spreading [8], [14]. The locations where filopodia initially adhered to substrates often direct the position of subsequently formed cell-matrix adhesions within the lamellum [19]–[23]. Cells thus take advantage of the filopodia as “sticky fingers” to explore their surroundings [1]–[17], [19]–[23], which consequently requires that filopodia are able to develop considerable tensile forces by which they pull on their environments [8], [22]–[24]. Tensile forces will either immediately rupture or further stabilize filopodia adhesions: the temporal stability of a filopodium increases once a contact with the extracellular matrix has been formed, and is further maintained when cells pull on their substrates [2], [3], [5], [8], [22]–[24]. In contrast, those subset of filopodia that fail to establish stable interactions with the extracellular matrix usually bend, move along the cell edge, and fuse with neighboring filopodia, often to be recycled back into the cell lamellum [21], [25]. The presence of tensile forces acting on filopodia adhesions is also reflected by the recruitment of certain force-regulated adaptor and scaffold proteins: the integrin containing cell-matrix adhesions within the filopodia shaft recruit talin and paxillin, VASP, but also vinculin, tensin and even zyxin [16], [20]–[22]. This is important to note since vinculin recruitment to talin requires the stretching of talin [26], [27]. As a consequence, the vinculin, tensin and zyxin adapters are typically found in mature focal adhesions localized in lamella but not to lamellipodia [28], [29], since adhesions maturation depends on myosin II driven tensile forces only once entering the lamellum [30]–[34]. Finally, filopodia are known to contribute to the assembly of contractile bundles and substrate adhesions in the lamella [21], but how this process is influenced by the lamellipodia dynamic is not well understood.

Although never addressed in the case of filopodia shaft adhesions, it has been demonstrated that the maturation of focal adhesions in the lamellum, is a myosin II-dependent process [35], but not that of nascent adhesions in the lamellipodium [36], and that focal adhesion maturation is coupled to the cycles of lamellipodium protrusions and retractions [37]. The lamellipodium exhibits cycles of protrusions and retractions, whereby the protrusions are driven by the actin polymerization of the dendritic actin network at the leading edge of the lamellipodium [38] while the retractions are initiated by actin-myosin II located in the lamellum just behind the lamellipodium-lamellum transition zone [35], [37], [39]. The formation and dynamics of filopodia and lamellipodia are regulated by different GTPases [40]–[44], suggesting that the formation, maturation and turnover of the adhesions associated with these two types of cell edge protrusions serve different purposes.

In fish fibroblasts, the appearance of filopodia adhesions, as recorded by paxillin recruitment, was observed to coincide with the advancement of the lamellipodium up to and past the respective adhesive segment of the filopodium [21]. In keratinocytes, when reached by the advancing lamellipodia, nascent filopodia adhesions, visualized via different cytoplasmic adaptors and proximity to the substrate, increase their size along the former orientation of filopodia resulting in more matured substrate adhesions [22], a maturation process which has been proposed to be associated with increased traction forces [32].

To understand the kinetic and mechanical aspects of how filopodia shaft adhesions grow and mature, and how this synchronizes with the protrusion of lamellipodia, we decided to visualize the formation of filopodia adhesions with respect to the clustering of β3-integrin receptors in the filopodia membrane. The β3-integrin is one of the major transmembrane proteins in the formation of earliest cell-substrate adhesions [28], [45]. While α5β1 integrins specifically recognize fibronectin (FN) and the clustering of α5β1 integrins determines the strength of cell-substrate adhesions [46], αvβ3 integrins mediate the rigidity sensing at the cell leading edge [47] and, together with talin, enable early mechanotransduction events [46]. To better understand how filopodia shaft adhesions are formed and get incorporated in the lamellum, we monitored the substrate adhesion and spreading of rat embryonic fibroblasts (REF52) that were stably transfected with β3-integrin-EGFP. This β3-integrin-EGFP construct has previously been validated carefully and utilized to study the kinetics of integrin clustering [45], [48], [49], as well as for studies detailing the mechanism of how the interaction of talin with β3-integrin enables talin activation and reciprocal integrin clustering [50]. Here we were particularly interested in characterizing the growth dynamics of filopodia shaft β3-integrin clusters in response to the local dynamics of protrusion and retraction of lamellipodia. In addition, the impact of lamellipodia dynamics on the characteristics of cytoskeleton organization at filopodia adhesion was examined. Since the cycles of lamellipodium protrusion and retraction are more pronounced on rigid than on soft polyacrylamide gels coated with FN, we finally asked how these different cyclic motions of lamellipodia due to substrate rigidity might affect the life cycle of filopodia shaft adhesions.

## Results

### Elongation and growth of β3-integrin-EGFP filopodia shaft adhesions is promoted in proximity to an advancing lamellipodium

**Figure 1:**
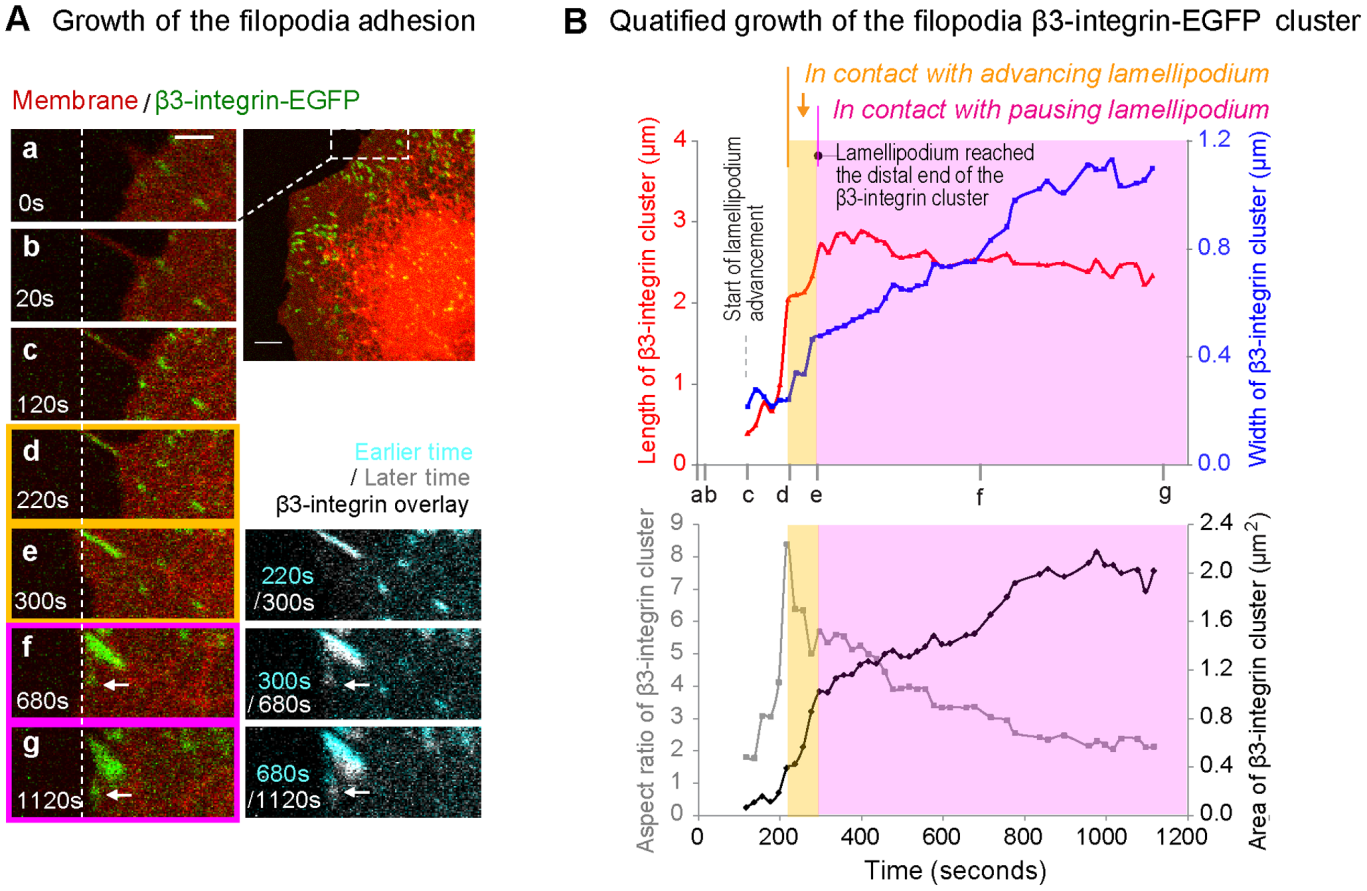
Growth kinetics of filopodia adhesions of transfected REF52 fibroblasts expressing β3-integrin-EGFP on FN coated glass. **A.** Selected frames from a confocal microscopy time-lapse sequence of a growing β3-integrin-EGFP cluster (green) (44 min–61 min after plating the cell, Video S1). The cell membrane was visualized by the membrane stain DiI (red). The dashed rectangle (top of the right cell image) was magnified in the left column (a–g). A rapidly protruding filopodium (a–b) forms a surface contact with the filopodium tip as seen by the initiation of β3-integrin-EGFP cluster (c). The white dashed vertical line indicates the initial tip position of the filopodia adhesion site. The colored frames in A correspond to the colored phases as classified in B, i.e. the time periods in which the β3-integrin-EGFP cluster was sequentially in contact with the advancing lamellipodium (yellow) and with the pausing lamellipodium without net advancement (pink) respectively. On the right bottom, the β3-integrin-EGFP images from different time points were overlaid (cyan-early, white-later). White arrows point to the newly formed β3-integrin-EGFP cluster at the side of the filopodia adhesion. **B.** Quantification of the growth kinetics of the filopodia β3-integrin-EGFP cluster and the associated filopodium. The letters (a–g) marked the time of the corresponding images (A, a–g). To indicate the movements of the lamellipodium when it is in contact with the growing filopodia β3-integrin adhesion, the plots were color-coded into zones corresponding to the color framed images as defined in A. The plots present the quantification of the filopodia β3-integrin-EGFP cluster characteristics, including its width (blue), length (red), area (black) and aspect ratio (grey). Dotted vertical grey line: the earliest time point that the lamellipodium started to advance toward the nascent filopodia integrin cluster. Scale bars: 2 µm (A, magnified views), 5 µm (A, overview).

To capture and quantify the kinetics by which filopodia shaft adhesions form, mature and get incorporated in the lamellum, REF52 fibroblasts stably transfected with β3-integrin-EGFP (REF52-β3-integrin-EGFP) were allowed to adhere and spread on fibronectin coated glass slides. By quantitative analysis (see methods) of time-lapse confocal fluorescence microscope sequences, we tracked the growth kinetics of β3-integrin filopodia adhesions. Several sequential growth phases of filopodia adhesion could be distinguished, providing information on how filopodia adhesions grow and get integrated into the cell lamellum (Fig. 1, Video S1).

#### Phase 1: Filopodia β3-integrin shaft adhesions rapidly elongated rearward towards the cell edge (Fig. 1A, a–d; 1B, white zone)

Shortly after the rapid protrusion of a filopodium (∼80 nm/s for ∼1 min (Fig. 1A–a, b)), we often observed the formation of a first surface contact as indicated by the occurrence of a green integrin cluster close to the filopodium tip (Fig. 1A–c). Immediately thereafter, the lamellipodium at the filopodium base started to advance along the filopodium shaft towards this filopodia integrin cluster (Fig. 1A, c–d). During the advancement of the lamellipodium, the length of the filopodium integrin shaft adhesion increased rapidly (Fig. 1B, red curve in white zone), while the width of the integrin adhesion remained unchanged (Fig. 1B, blue curve in white zone). The resulting rapid increase of the aspect ratio of the filopodia shaft adhesion is given as well (Fig. 1B, grey curve in white zone).

#### Phase 2: Rearward growing filopodia β3-integrin shaft adhesions are often overrun by advancing lamellipodia (Fig. 1A-d, e; 1B, yellow zone)

For the filopodium shown, the advancing lamellipodium reached the proximal end of the filopodia integrin adhesion 120 s after the filopodia adhesion was initiated (Fig. 1A–d). It took about additional 80 s for the lamellipodium to advance across the filopodium shaft adhesion and reach its distal end (Fig. 1A–e). This resulted in the full entry of the filopodium adhesion into the lamellipodium. In this period, the depicted filopodium adhesion increased its width, while the length further increased reaching its maximal length.

#### Phase 3: Integration of the filopodia β3-integrin shaft adhesions into pausing lamellipodia (Fig. 1A–e, f, g and their overlays; Fig. 1B, pink zone)

After reaching the distal end of a filopodium β3-integrin shaft cluster, the lamellipodium typically paused at this position without net advancement for the rest of the tracked duration. Correspondingly, the filopodia β3-integrin adhesions that integrated into lamellipodia did not grow further in length, but continued to widen, causing a rapid decrease in their aspect ratio while their total area further increased. Here after 600 s of growth, the depicted filopodium shaft adhesion had significantly increased in width and area. New β3-integrin clusters (Fig. 1A, white arrows) also started to form at their sides and eventually merged with the sidewise growing filopodia adhesions.

**Figure 2:**
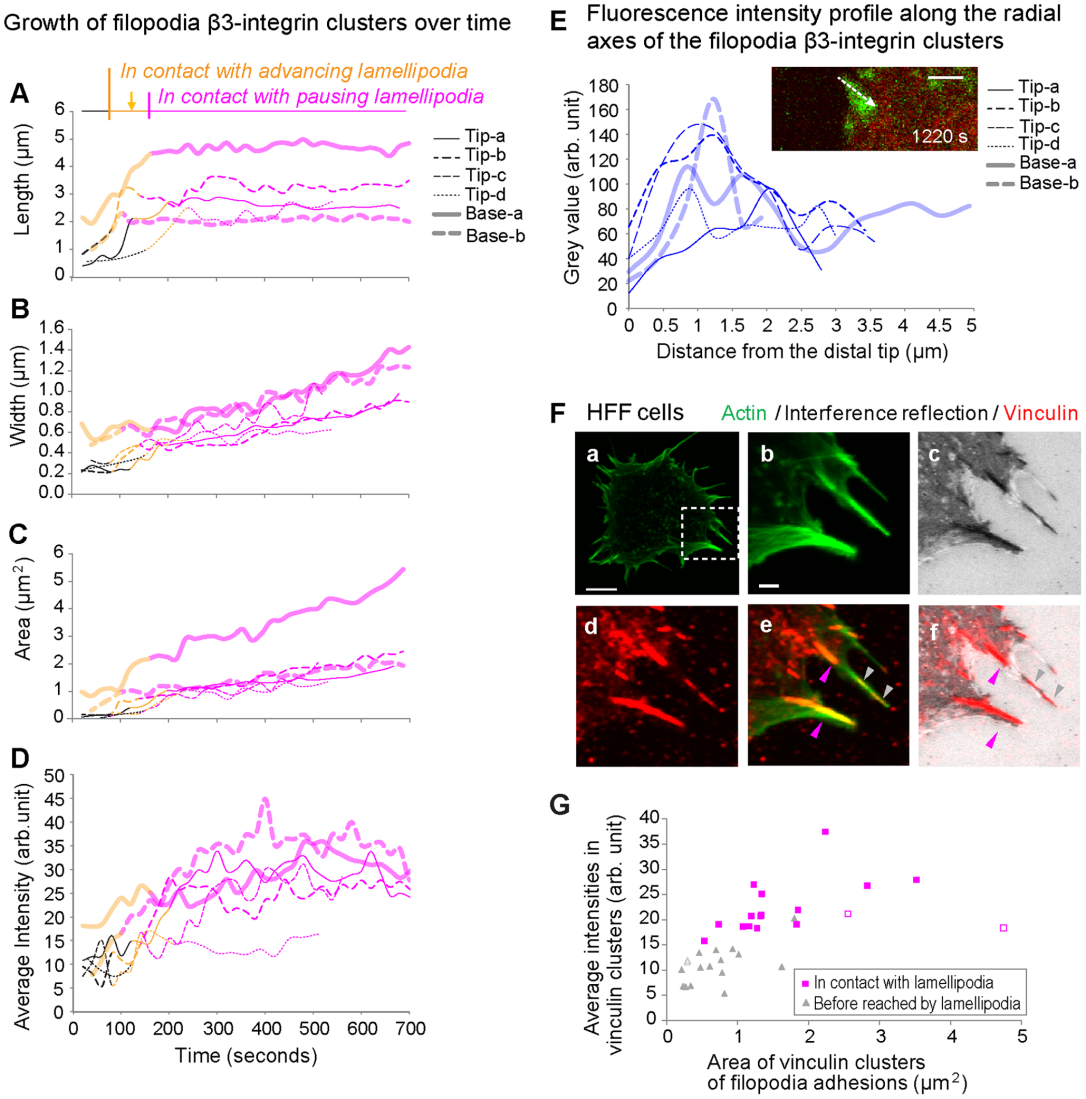
Growth kinetics of β3-integrin-EGFP clusters of filopodia tip or base adhesions (A–D.). Each curve represented one filopodia adhesion as REF52-β3-integrin-EGFP cells spread on FN coated glass (cells after plating, 44–56 min (Tip a), 12–24 min (Tip b), 17–29 min (Tip c), 25–37 min (Tip c), 5–17 min (Base a), 7–19 min (Base b)). The length (A), width (B), area (C) and average fluorescence intensity (D, average = sum of grey value/number of pixels) were plotted for β3-integrin-EGFP clusters for filopodia tip adhesions (thin curves, representing 4 maturing filopodia adhesions in 3 cells) and filopodia base adhesions (thick opaque curves, representing 2 maturing filopodia adhesions in 1 cell). Curves Tip-a and Base-a corresponded to the filopodia adhesions shown in Fig. 1 and Fig. S1 respectively. The traces were color coded (similar to the colored zones in Fig. 1B) to indicate the different local movements (advancing or pausing) of the lamellipodium in relation to the growth of the filopodia adhesion (*black*: before being reached by lamellipodium; yellow: being passed by an advancing lamellipodium; *pink*: with the lamellipodium persisted at the distal end of the filopodia adhesion without net advancement). **E.** The spatial distribution of β3-integrins in filopodia adhesions. For the filopodia adhesions analyzed in A–D, the fluorescence intensity profiles were generated along the long axes of filopodia β3-integrin-EGFP clusters in the last frame of their tracked sequences, as indicated by the dashed arrow in the *Insert* (same as analyzed in Fig. 1; green: β3-integrin-EGFP; red: membrane). The range of the x axis for the intensity profile curves corresponded to the full lengths of filopodia adhesions between their distal and proximal ends. **F.** Vinculin recruitment to filopodia adhesions. The white squared cell edge region of a HFF cell (a, 20 min plated on FN coated glass) was magnified (b–f) in respective signals and their overlays. **G.** Scatter plot of average fluorescence intensities of vinculin clusters within filopodia adhesions with respect to their areas. The grey and pink data points corresponded to the filopodia adhesions before reached by lamellipodia or in contact with lamellipodia respectively. Open symbols correspond to the filopodia adhesions indicated by the arrowheads (in respective colors) in e and f. Scale bars: 2 µm (F–b, E insert), 10 µm (F–a).

While the above analysis was based on the β3-integrin adhesions initiated at the distal tip of the filopodium (filopodia tip adhesions), similar β3-integrin adhesions could also be formed at the bases of filopodia (filopodia base adhesions), which were in contact with lamellipodia from the beginning of their initiation (Fig. S1; Video S2). Importantly, comparable sequences of events were observed for the maturation of filopodia shaft adhesions in REF52 and human foreskin fibroblast (HFF) cells (Figs 1, 2A–C, S1A–C, S4A,B). Upon maturation, filopodia adhesions generally existed for more than 10 minutes (Fig. 2A–C), with some over 20 minutes (Fig. S1A, B). Furthermore, in cases where the filopodia had a non-adherent distal section, these filopodia tips deteriorated (bending, fracturing, recycling or lateral movement (Figs S1A, S4A; Video S2)) as observed previously in fish fibroblasts [21]. However, these movements of non-adherent distal filopodia tip segments did not alter the growth characteristics of more proximal filopodia adhesions (Fig. S1A, B, black arrows). Overall, the growth characteristics of filopodia adhesions were distinctively different from point-like nascent adhesions formed at the lamellipodium type of cell edge ([36], Fig. S5).

### Time-dependent quantification of β3-integrin-EGFP intensity and vinculin recruitment

To quantify the fluorescence intensity of filopodia β3-integrin-EGFP clusters as a function of time (Fig. 2D), the average fluorescence intensity per pixel of filopodia β3-integrin-EGFP clusters was measured. It rapidly increased when first contacting the advancing and later the pausing lamellipodium (Fig. 2D, yellow and pink segments respectively). Shortly after the onset of the lamellipodial pausing, the fluorescence intensity reached a plateau, and then slightly declined due to photobleaching.

To quantify the impact of the pausing lamellipodium, we analyzed the spatial densities of β3-integrins in filopodia adhesions as probed by the fluorescence intensity profile (Fig. 2E) along the long axis of the filopodia β3-integrin-EGFP cluster (Fig. 2E, insert) at the end of its tracked duration. For the steadily maturing filopodia adhesions, the fluorescence intensity of β3-integrin-EGFP clusters peaked at a distance of 0.9±0.6 µm (n = 8, 4 cells) away from the distal ends of filopodia adhesions, and then decreased towards their proximal ends.

Since vinculin recruitment to newly formed integrin adhesions requires force-activation of talin or α-actinin [26], [27], [30], [31], [51]–[53], with a close correlation of force and vinculin recruitment to focal adhesions in live HFF cells [54], we immunostained for vinculin in HFF cells 20 min after cell seeding (Fig. 2F). In agreement with the literature [20], [22], we see that vinculin gets recruited not only to focal adhesions, but importantly also to the filopodia shaft adhesions already prior to their integration into the lamellum (Fig. 2F). Here we find that vinculin recruitment to filopodia shaft adhesions was upregulated once they got in contact with the lamellipodium (Fig. 2F). The average (per pixel) intensities, as well as the area, of recruited vinculin in the filopodia adhesion in contact with a lamellipodium (Fig. 2F, pink arrowheads) were significantly higher than those of the filopodia shaft adhesions located in front of a lamellipodium (Fig. 2F, grey arrowheads, Fig. 2G). In addition, the vinculin staining fully colocalized with the dark regions in the interference reflection image (Fig. 2F–c, f), indicating a tight surface anchorage of these filopodia adhesions to the substrate. This suggests that tensile forces are already applied to the filopodia shaft adhesions, and that the force-activated vinculin recruitment is upregulated once filopodia shaft adhesions get integrated into the lamellipodium.

### Lamellipodia undergo cyclic protrusions and retractions in the proximity of filopodia adhesions

**Figure 3:**
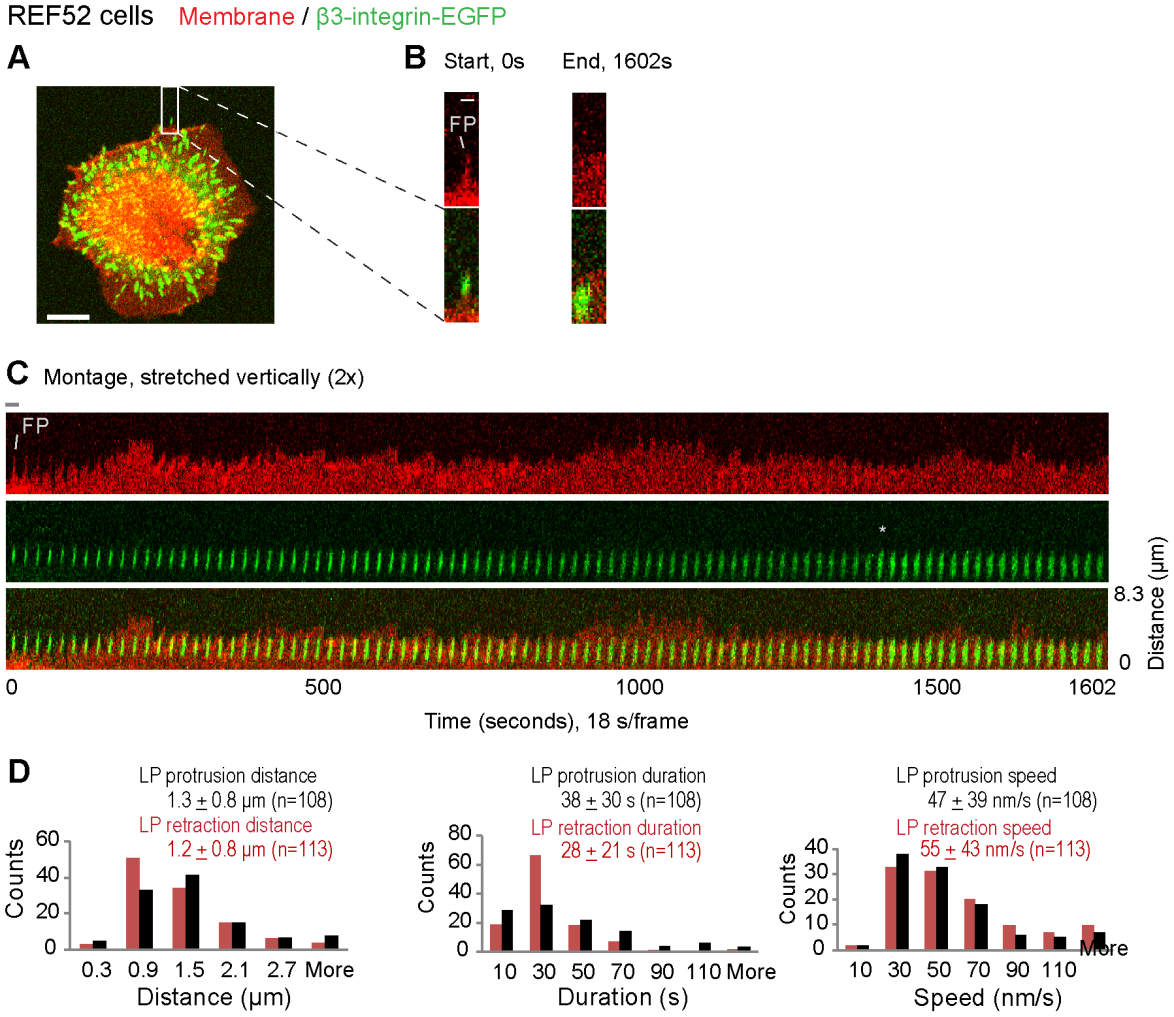
Cycles of periodic protrusions and retractions of a lamellipodium in the proximity of maturing filopodia adhesions. **A.** The β3-integrin-EGFP (green) expressing REF52 fibroblast on a FN coated glass surface was time-lapse tracked with confocal microscopy. The cell membrane was stained by the fluorophore DiI (red). **B.** Magnified views of the white rectangle region in A showed the filopodia adhesion at the start (left) and end (right) of the time-lapse tracking sequence (20 min–47 min after seeding, Video S3). **C.** Time-lapse montages of the white rectangle region in A. At the time point indicated by the grey asterisk, we had to increase the laser (488 nm) intensity to compensate for photobleaching. To better visualize lamellipodium activities, the presented montage was stretched vertically 2×. **D.** Histograms of the distances, durations and speeds of the cyclic protrusions (black bars) and retractions (brown bars) of lamellipodia in proximity to filopodia adhesions. Values in the parenthesis gave the number of measurements (at 14 filopodia adhesions in 5 cells). Scale bars: 1 µm (B), 10 µm (A). The horizontal grey bar indicates the width of a single image frame in the montage in C (2.5 µm).

Previous studies of spreading cells [14], [35], [37], [55] have shown that lamellipodia undergo cycle of protrusion and retraction in fibroblasts adhering to rigid surfaces [37]. To examine the dynamics of lamellipodia at filopodia adhesions, we analyzed the confocal fluorescence time-lapse data of the REF52-β3-integrin-EGFP cells plated on FN coated glass (Fig. 3). The dynamics of the lamellipodium movement were monitored by DiI fluorescent staining of the cell lipid membrane (red) with respect to the filopodia β3-integrin adhesion (green). As demonstrated by the fluorescence montage of the cell edges, periodic lamellipodia protrusions and retractions occurred in proximity to filopodia β3-integrin adhesions even after their integration into the lamellipodium was completed (Fig. 3C, Video S3). To better visualize the protrusions and retractions of lamellipodium in the vicinity of a filopodia adhesion, we generated kymographs at multiple positions along the cell edge close to the filopodia β3-integrin adhesion (Fig. S2A). Comparison of corresponding kymographs demonstrated that the spatio-temporal patterns of the periodic protrusion and retraction cycles of the lamellipodium remained similar in the vicinity of filopodia β3-integrin adhesions (18 filopodia adhesions in 6 cells, see e.g. Fig. S2A–b), and that the kinetics are similar for filopodia-containing cell edges in both spreading REF52 cells and HFF cells on FN coated glass (Fig. S2B, D). For REF52 cells, the leading edge protruded on average for 38±30 s and then retracted for 28±21 s., while the average distances traveled by lamellipodia during their protrusions and retractions were 1.3±0.8 µm and 1.2±0.8 µm respectively (Fig. 3D). It should be noted that these similarities between REF52 and HFF cells are expected since the molecular modules used by different fibroblasts and by many other cells, by which integrins are coupled to the actin cytoskeleton, are the same.

### Inhibition of myosin light chain kinase suppresses the growth of the filopodia shaft adhesions

**Figure 4:**
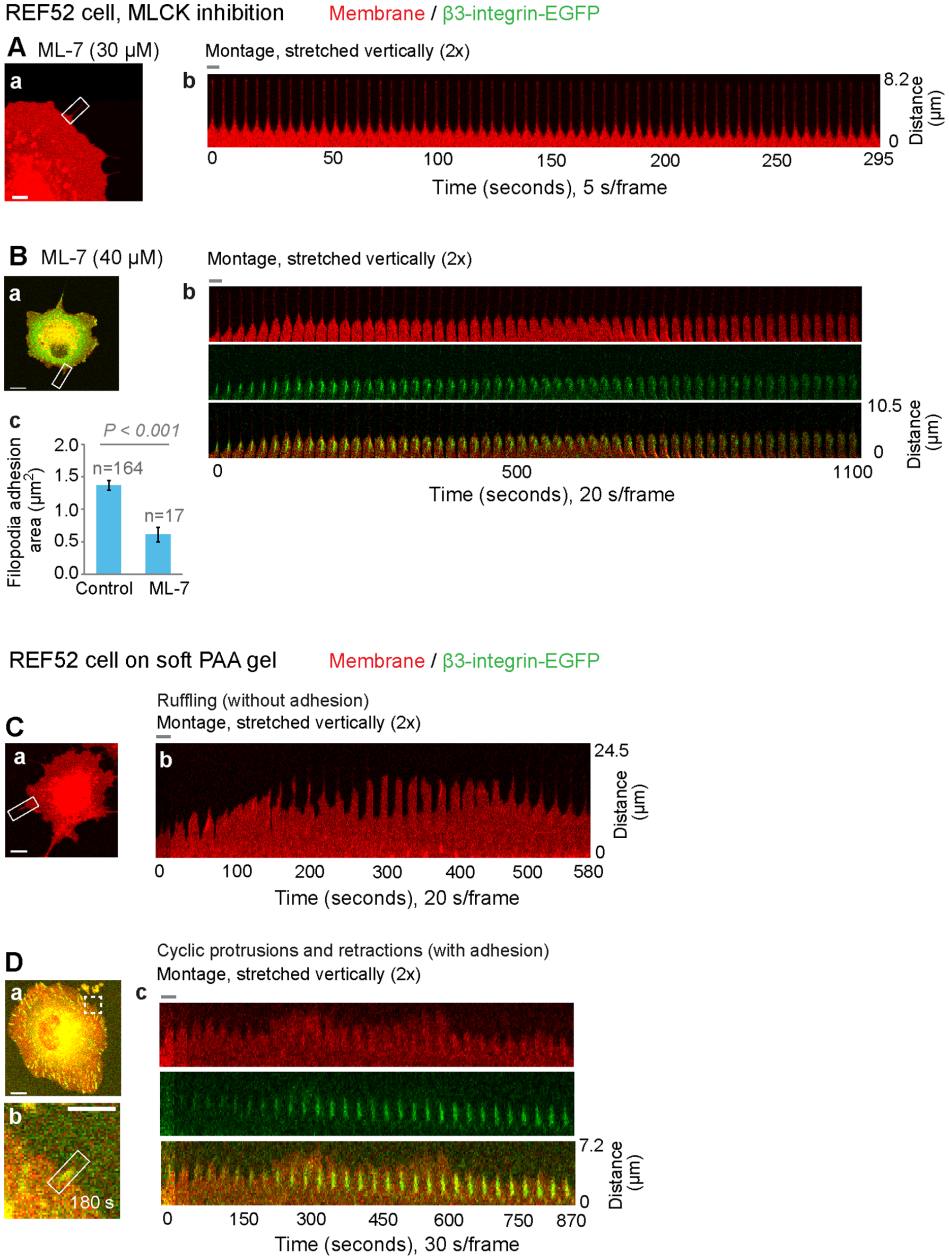
Growth kinetics of filopodia adhesions after MLCK inhibition (A, B) or on soft polyacrylamide gels (C, D). The β3-integrin-EGFP (green) expressing REF52 fibroblasts on FN coated glass (A, B) or polyacrylamide gel (C, D) surfaces were time-lapse tracked with confocal microscopy. The cell membrane was stained by the fluorophore DiI (red). **A and B.** Inhibition of MLCK by ML-7 suppressed the cyclic protrusions and retractions of lamellipodium and the growth of filopodia adhesions. **A.** Time-lapse montage (b) of the filopodium containing cell edge region (white rectangle, a) showed the loss of the cyclic protrusions and retractions of the lamellipodium in the proximity of the filopodium in ML-7 treated cells (25 min–30 min after plating, Video S4). **B.** Time-lapse montage (b) of the filopodia adhesion cell edge region (white rectangle, a) demonstrated the suppressed size growth of the filopodia β3-integrin adhesion in ML-7 treated cells. c. Comparison of the area (average of the tracked temporal states (0–600 s)) of the filopodia β3-integrin adhesions (7 adhesions, n = 17) in the ML-7 (30 or 40 µM) treated cells with that of the maturing filopodia β3-integrin adhesions (6 adhesions, n = 164) in the cells plated in normal culture media. Error bars correspond to the standard errors. **C and D.** The growth of filopodia adhesions in REF52-β3-integrin-EGFP cells on soft PAA substrates was associated with the restored cyclic protrusions and retractions of lamellipodia. The PAA substrate (7.4 kPa) was covalently coated with FN. **C.** Cell without stable surface adhesions (a, 103 min–113 min after plating, Video S5) exhibited significant ruffling as shown by the time-lapse montage of the cell edge (b). **D.** The filopodia adhesion (white rectangle, b) at the edge of a cell (white square in a, 121 min–136 min after plating, Video S6) matured in association with the restored cyclic protrusions and retractions of the lamellipodium (c). To better visualize lamellipodium activities, all montages were stretched vertically 2×. Scale bars: 5 µm (A, D–b), 10 µm (B, C, D–a). The width of a single image frame in the montage is indicated by the horizontal grey bar: A–b, 2.5 µm; B–b, 4.5 µm; C–b, 6.2 µm; D–c, 3 µm.

Myosin II is concentrated behind the lamellipodium/lamellum transition zone [56] and the activation of myosin II by myosin light chain kinase (MLCK) was shown to be necessary for the periodic contractions of lamellipodia [35], [37]. We thus biochemically inhibited MLCK in spreading REF52-β3-integrin-EGFP cells using the specific inhibitor ML-7. As predicted, this treatment inhibited the cyclic protrusions and retractions of the lamellipodia (5 s/frame) (Fig. 4A, Video S4), and the edges of ML-7 treated cells were dominated by lamellipodia (Fig. 4B–a). Far fewer filopodia were seen in ML-7 treated cells and the few that formed had filopodia β3-integrin adhesions of considerably suppressed growth (n = 16) (Fig. 4B–b), and a significantly smaller size (0.61±0.25 µm^2^, p<0.001) when compared to maturing filopodia β3-integrin adhesions in untreated cells (1.37±0.95 µm^2^, Fig. 4B–c). Therefore, our data suggested that the acto-myosin activity is required for the maturation of filopodia shaft adhesions.

### Filopodia adhesions stabilize lamellipodia and thereby enable their local cyclic protrusions and retractions on soft polyacrylamide gel

Since cyclic protrusions and retractions of lamellipodia were previously shown to occur only on rigid, but not on FN-coated soft polyacrylamide (PAA) gels [37] where the cell adhesions were found to be more diffuse and unstable [57], we studied the spreading of REF52-β3-integrin-EGFP cells on soft (7.4 kPa) PAA gels covalently coated with FN. Due to the lack of stable adhesion of the cell edge to soft substrate, the lamellipodium along the cell periphery demonstrated significant ruffling (Fig. 4C–b, Video S5), which is in agreement with the previous literature [37]. However, filopodia shaft adhesions could form on soft PAA substrates and were integrated into lamellipodia, which coincided with a reduction in lamellipodial ruffling activity (Fig. 4D–c, Video S6, 19 adhesions in 3 cells). The presence of a filopodia shaft adhesion thus locally restored the cyclic protrusion and retraction activities of the lamellipodium. Such filopodia adhesions on soft PAA (7.4 kPa) exhibited growth characteristics (Fig. S3) similar to those observed on rigid (glass) surfaces, as well as on PAA substrate with intermediate stiffness (35 kPa), where lamellipodia also exhibited suppressed ruffling activity at sites of filopodia shaft adhesions.

### Reduced contact time between filopodia shaft adhesions and advancing lamellipodia blocks their maturation

**Figure 5:**
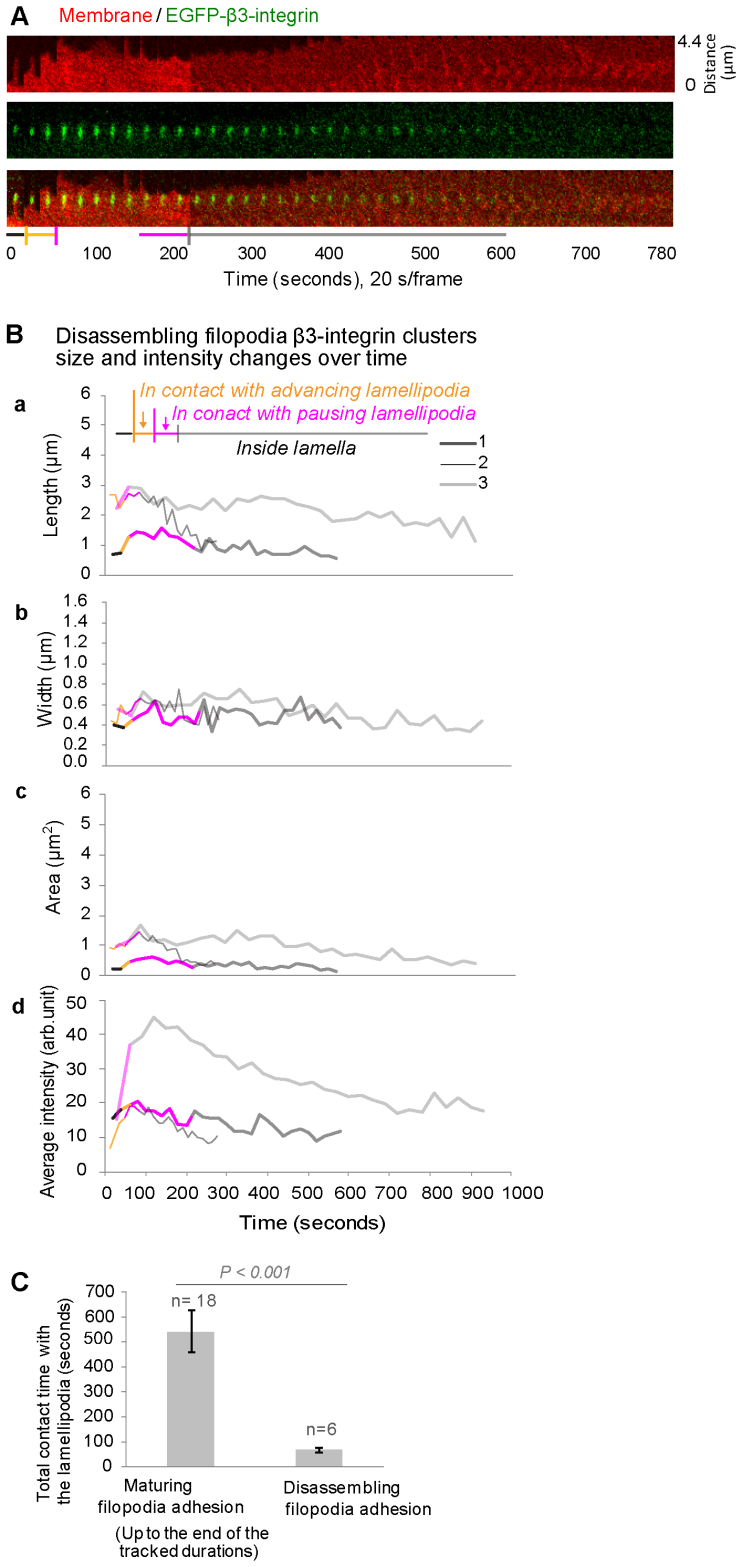
The suppressed growth of filopodia adhesions in cases of insufficient contact time with the lamellipodium. **A.** Time-lapse montage of the cell edge region containing a disassembling filopodia adhesion in a β3-integrin-EGFP (green) expressing REF52 fibroblast on FN coated glass (40 min–53 min after plating, Video S7). This dissembling filopodia adhesion was located in the same cell region and captured in the same image sequence as for the maturing filopodia adhesion analyzed in Fig. 1. The cell membrane was stained by the fluorophore DiI (red). The colored bars indicate the relation between the filopodia adhesion and the lamellipodium as in Fig. 2A. *Black segment:* filopodia adhesion before being reached by the advancing lamellipodium. *Yellow segment*: passing of the advancing lamellipodium. *Pink segment*: pausing of the lamellipodium at the distal end of the filopodia adhesion without net advancement. *Grey segment:* the disassembly of the filopodia adhesion inside the cell lamellum after its full separation from the advancing lamellipodium. **B.** Time traces of the length (a), width (b), area (c) and average fluorescence intensity (d) of the disassembling filopodia β3-integrin clusters (same color code as for the maturing filopodia adhesions in Fig. 2A–D). Growth trace 1 corresponds to the dissembling filopodia adhesion in A. **C.** Comparison of the total contact time with lamellipodia (the averaged sum of the advancing and pausing durations of lamellipodia at filopodia adhesions) between the disassembling filopodia adhesions (n = 6) and those matured into stable adhesions (n = 18). Error bars correspond to the standard errors.

Though less often observed at our highest spatiotemporal resolution, some filopodia shaft adhesions (<10%) rapidly disassembled after their formations. One of such filopodia β3-integrin shaft adhesions (Fig. 5A, Video S7) was located at a short filopodium (∼2 µm in length). Typically, it took less than 20 s (yellow horizontal bar) for the advancing lamellipodium to move across such filopodia shaft adhesions. After briefly pausing (∼60 s, pink horizontal bar) at its distal end, lamellipodia resumed their advancement and left the filopodia adhesion behind in the cell lamellum (grey horizontal bar). The β3-integrin clusters of these filopodia adhesions were small and disassembled rapidly. This was in drastic contrast to the long stability of maturing filopodia shaft adhesions that were in contact with a pausing lamellipodium as analyzed earlier (Fig. 1).

The failed growth of these filopodia adhesions was further quantified by monitoring the growth traces over time with respect to the area, length, width, and average fluorescence intensity of their β3-integrin-EGFP clusters (Fig. 5B). Although they started with similar sizes as compared to the later maturing filopodia shaft adhesions (Fig. 2A–C), their growth was suppressed (trace 3) or they rapidly disintegrated (traces 1 and 2) (Fig. 5B–a, b, c). Similarly as compared to the maturing filopodia adhesions (Fig. 2D), the fluorescence intensities of the disassembling filopodia β3-integrin-EGFP clusters had temporally increased during their brief contact with lamellipodia (Fig. 5B–d, yellow and pink segments), but they rapidly decreased after the separation of the advancing lamellipodium from these filopodia adhesions. Importantly, all the observed unstable filopodia adhesions had very short total contact time with lamellipodia (Fig. 5B, yellow and pink segments), with an average duration of 69±47 s (Fig. 5C). This is significantly shorter than observed for the maturing filopodia adhesions (542±360 s, limited by the tracking durations, Fig. 5C), which stayed in contact with a pausing lamellipodia. Sufficient contact time with the local pausing lamellipodium thus seem to be critical parameter regulating the maturation of filopodia adhesions.

### Slow lamellipodium advancement increased the contact time and promoted the maturation of filopodia adhesions

**Figure 6:**
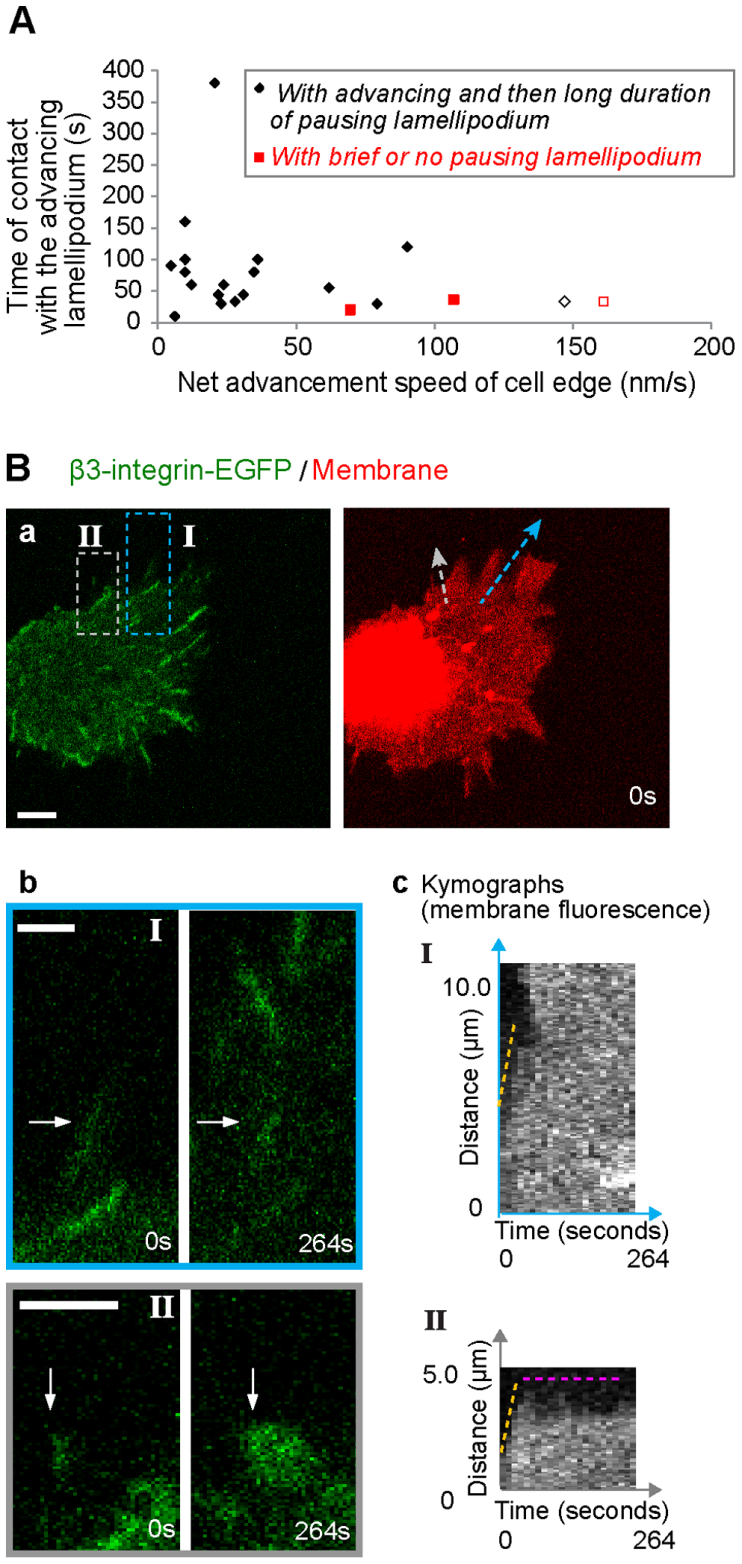
The effects of the net advancement speed of the cell edge on the contact time between the lamellipodium and a filopodia adhesion. **A.** Scatter plot of the part of the contact time of a filopodia adhesion with the advancing lamellipodium, with respect to the net advancement speed of the cell edge. All data were for REF52-β3-integrin-EGFP cells spreading on FN coated glass. *Black:* maturing filopodia adhesions. *Red:* disassembling filopodia adhesions. Due to the fast net advancement speed of the cell edge, the disassembling filopodia adhesions demonstrated a reduced contact time with the advancing lamellipodia, which subsequently had very brief of no pausing at these filopodia adhesions. Open symbols correspond to the filopodia adhesions in B–b. **B. a.** A fast spreading REF52 fibroblast expressing β3-integrin-EGFP (green) on FN coated glass (5 min–9 min 30 s after plating, Video S8). The cell membrane was stained with the fluorophore DiI (red). **b.** Magnified views of the dashed rectangle regions (I and II) in a. The selected frames from the time-lapse sequences showed the disassembly (I, horizontal arrows) or steady maturation (II, vertical arrows) of the filopodia adhesions as a result of ongoing advancement or prolonged pausing of lamellipodium at the two filopodia adhesions respectively. **c.** The advancement of cell two edges are represented by the kymographs that were generated along the corresponding kymograph lines (colored arrows in a) from the membrane fluorescence signal at the two filopodia adhesions. The dashed lines indicate the advancing (yellow) and pausing (pink) phases of lamellipodia at the respective filopodia adhesions. The net advancement speeds of the cell edges were measured at the corresponding sections of the kymographs as indicated by the dashed yellow lines (B-c I, 161 nm/s; B–c II, 147 nm/s). Scale bars: 2 µm (B–b), 5 µm (B–a).

Next, we quantified the net advancement speed of the cell edge. This speed at the filopodia adhesion was determined as Δd/Δt (net advancement distance of cell edge (Δd) divided by the total time of cell edge movement (Δt)) in the kymograph (Fig. S2C). The part of the contact time between filopodia adhesions and the advancing lamellipodia (e.g. Fig. 1, yellow zone) was plotted as function of this speed of the spreading REF52-β3-integrin-EGFP cells (Fig. 6A). Our data demonstrated a wide range of contact times (10–400 s) of filopodia adhesions with the advancing lamellipodia. The net advancement speed of the cell edge was below 40 nm/s for most of filopodia adhesions whereby the lamellipodia in all of these cases first advanced and then paused for long durations at the distal ends of the filopodia adhesions which steadily matured. Similarly in spreading HFF cells, we could observe a similarly wide range of cell edge advancement speeds (9–100 nm/s) and the long duration of positional pausing of the slowly advancing lamellipodia at the distal tips of filopodia adhesions (Fig. S4).

In summary, lamellipodia were often found to either pause at the distal ends of the filopodia shaft adhesions or to keep advancing (Fig. 6A, black and red data points respectively). This resulted either in their subsequent maturation or disassembly respectively, when the cell edge advanced too rapidly (>40 nm/s). These two modes of contact between the fast advancing lamellipodia and filopodia shaft adhesions, and the respective fates of the filopodia shaft adhesions, were observed in both REF52-β3-integrin-EGFP cells (Fig. 6B, Video S8) and HFF cells (Fig. S4C, D; Video S9, S10).

### Circumferentially oriented actin fibers intersect with former filopodia shaft adhesions within the cell lamella

**Figure 7:**
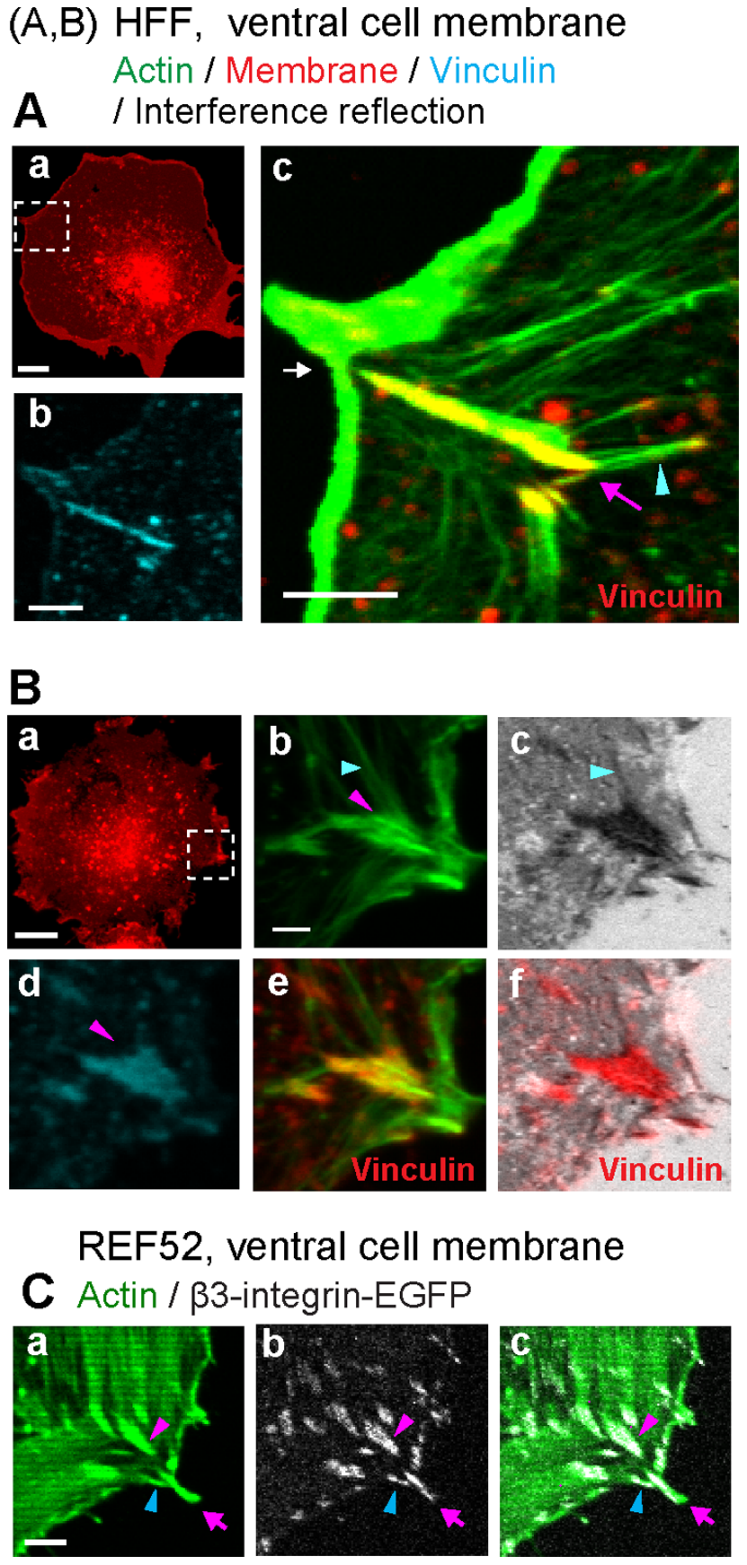
The actin cytoskeleton organization at filopodia adhesions as seen in ventral cell membrane samples. Cells were gently blasted open. While this might cause some local disruptions, the main purpose was to distinguish between molecular components that are weak versus strongly bound to the ventral site of the plasma membrane. The colors of stains denote the ventral cell membrane (red) and actin structures (green). Filopodia adhesions were identified (see method) by vinculin immunostaining (cyan, A–b, B–b), interference reflection signals (grey images, B), and β3-integrin-EGFP imaging (grey, C). **A and B.** Cell edge regions of the exposed ventral cell membranes from HFF cells (20 min (A) or 33 min (B) after plating on FN coated glass). The white square regions (A–a, B–a) were magnified respectively (b, c in A; b–f in B). In merged images, vinculin was alternatively false colored in red (A–c, B–e, B–f) for a better presentation of the colocalization of signals. Pink arrow or arrowheads denote the former filopodia actin bundles and their associated substrate adhesions. The cyan arrowheads indicate circumferential stress fibers with their surface anchorage (A) or tight surface association (B). The white arrow (A–c) indicates the separation between the filopodia adhesion in the cell lamellum and the distal section of the filopodium, which might be fracture that could have occurred during the sample preparation. **C.** The cell periphery of an exposed ventral cell membrane from a REF52-β3-integrin-EGFP cell (30 min after plating on FN coated glass) showed a β3-integrin cluster at the filopodium (pink arrows), circumferential stress fibers (cyan arrowheads) anchored to the substrate via the β3-integrin clusters formed at the side of this filopodia adhesion, and the sidewise widening of the filopodia β3-integrin cluster (pink arrowheads) following the connected thick circumferential stress fibers in the cell lamellum. Scale bar: 5 µm (A–b & c, B, C), 10 µm (A–a).

Finally, to examine the actin cytoskeleton organization at filopodia adhesions, we used a newly developed ventral cell membrane preparation method which allowed us to expose the partially well-preserved ventral cell membrane with its associated cell-substrate adhesions and actin structures. During the cell burst, only those actin filaments that are firmly anchored to the ventral membrane remain attached. Figure 7 shows that, in adherent REF52-β3-integrin-EGFP and HFF cells, the former filopodia shaft adhesions (Fig. 7, pink arrows and arrowheads) were connected to the circumferentially (in parallel to the cell edge) oriented actin stress fibers of varied thickness (Fig. 7, cyan arrowheads). These fibers were located in the ventral cell membranes, and often in tight contact with the substrate surface. Filopodia adhesions also retained the radial (toward the cell center) oriented original filopodia actin bundles located in ventral cell membranes (Fig. 7, actin images, pink arrows and arrowheads). While the newly formed filopodia adhesions fully colocalized with the slender filopodia actin bundles (Fig. 7A,C, pink arrows), more developed filopodia adhesions with sidewise widened β3-integrin clusters were associated with the sidewise deformed filopodia actin bundles (Fig. 7B,C, pink arrowheads).

## Discussion

Here we describe for the first time the kinetics how the maturation of filopodia shaft adhesions correlates with the cyclic protrusion and retraction activities of the lamellipodium. While filopodia adhesions were previously found to be integrated into the cell lamella of various cell types [20]–[22], [32], [58], no information was available about the integration process. Here we show that filopodia shaft adhesions grow and mature in sequential phases that correlate with the advancing and pausing of the adjacent lamellipodium (Figs 1, 2, S1). We also show that vinculin gets recruited into the filopodia shaft adhesions (Figs 2F, 7A), which suggests that they are exposed to tensile forces. In contrast to the previously described behavior of nascent adhesions within lamellipodia [36], which remain point-like and can be turned-over rapidly [36], the β3-integrin-rich filopodia shaft adhesions once formed increase rapidly in length and their initial formation typically induces the advancement of the proximal lamellipodium (phase 1) (Figs 1, 2A, S1; Video S1). In the next phase, the rearward growing filopodia shaft adhesions are overrun by the advancing lamellipodium (phase 2) until the point, where the lamellipodia pauses at the distal edges of filopodia shaft adhesions (Figs. 1, S1; Video S1, S2). Once lamellipodia started to pause, the now integrated filopodia shaft adhesions started to grow in width (phase 3) (Figs 1, 2A, 2B, S1).

Previous studies have shown that the activity of myosin II just behind the lamellipodium-lamellum transition zone regulate the cyclic retraction of the lamellipodium [24], [35], [37], [59] and the maturation of cell-matrix adhesions and substrate rigidity sensing [35], [37]. Our work could show now that the maturation of filopodia adhesions and their integration into the lamellum are also regulated by tensile forces. This was suggested by the upregulated recruitment of vinculin (Fig. 2F,G), which only binds to stretched but not relaxed talin [26], [27], and the temporal increase of integrin fluorescence in filopodia shaft adhesions (Fig. 2D) once reached by the advancing lamellipodium, and by the existence of the cyclic protrusions and retractions of lamellipodia in the proximity of filopodia adhesions on FN-coated rigid (Figs 3A, S2A) and soft (PAA, Fig. 4D) surfaces. MLCK inhibition blocked the periodic protrusions and retractions of the lamellipodium (Fig. 4A,B) as previously described [37]. But MLCK inhibition did not perturb the initial formation of filopodia adhesions and their subsequent incorporation into lamellipodia, probably because the leading edges of cells are pushed forward by the protrusive forces generated by the barbed end elongation of the dendritic actin network [38]. Interestingly, the spatial distribution of the fluorescence intensity in the mature filopodia β3-integrin-EGFP cluster typically revealed a characteristic intensity maximum at about 1–1.5 µm away from the distal tip of the filopodia adhesion (Fig. 2E). This distance is similar to the often described distance from the cell edge to the lamellipodium-lamellum transition [29], [60]–[62]. These further suggested the maturation of the filopodia β3-integrin-EGFP cluster is coupled to the cycles of myosin II-generated forces.

The stability and lateral growth of β3-integrin-EGFP clusters originating from filopodia shaft adhesions (Figs 2A–C, 5B, S3) are shown here to be dependent on the contact time with the periodically advancing and retracting lamellipodium (Fig. 5C). Filopodia shaft adhesions mature upon their integration, but only when the local cyclically retracting lamellipodium paused its net advancement for a long duration at their distal tips (Figs 1, 2, 3, S1, S3). In contrast, the disassembled filopodia adhesions exhibited only short contact time with lamellipodia (69±47 s, Fig. 5C), which occurred often when lamellipodia edge rapidly passed filopodia adhesions (Figs 5; 6B–b, S4C). Alternatively, when the cyclic protrusions and retractions of lamellipodia did not occur (Fig. 4B), filopodia adhesions failed to mature. Particularly in the former case, the contact time between lamellipodia and the disassembling filopodia adhesions (Fig. 5C) is similar to the time period of the lamellipodia retraction cycles (Fig. 3D), suggesting that maturation or reinforcement did not take place during the lamellipodial advancement phase. Thus the contact time and multiplicity of retraction cycles of the lamellipodium at the filopodia adhesions regulates the maturation or disassembly of filopodia adhesions. Cyclic protrusions and retractions of lamellipodia have also been associated with the deposition of circumferential actin filaments that run parallel to the cell edge in various cell types [35], [55], [63] which became efficiently coupled with focal adhesions at the onset of the lamellum right behind the transition zone [64]. Our data now suggest that the circumferential actin filaments become connected with filopodia adhesions behind the lamellipodium-lamellum transition (Fig. 7).

To conclude, filopodia and lamellipodia both sense the mechanical aspects of a cell’s environment and their respective activities are key in the cell migratory behaviors, such as cell spreading [2], [7], [8], [14], [23], the path-finding of neuronal growth cones [65], [66], angiogenesis [12], as well as cancer cell migration and invasion [13]. Our work now highlights the previously unrecognized interplay between the maturation of filopodia shaft adhesions and their regulation by the cyclic advancing and retraction dynamics of the proximal lamellipodium.

## Materials and Methods

### Cell culture

REF52 cells, stably transfected to express β3-integrin with EGFP tag on its intracellular domain (Dr. Bernhard Wehrle-Haller, University of Geneva, Switzerland) [45], [48]–[50], were cultured in Dulbecco’s modified Eagle’s media (DMEM) (Gibco, Invitrogen) supplemented with 10% fetal calf serum (Gibco, Invitrogen) and 1% penicillin–streptomycin (Gibco, Invitrogen). HFF cells (PromoCell) were cultured in fibroblast growth medium (PromoCell). For experiments, the culture media for HFF cells were changed to the GlutaMAX-1 containing DMEM (Gibco, Invitrogen) supplemented with 10% newborn calf serum (Gibco, Invitrogen) and 1% penicillin–streptomycin (Gibco, Invitrogen). All cells were cultured in culture flask (BD Biosciences) in a humidified incubator at 37°C with 5% CO_2_ and 95% air for several passages before experiments.

### Substrate preparation

FN was isolated from human plasma (Zürcher Blutspendedienst SRK) using gelatin-sepharose chromatography based on established methods [67]. The Lab-Tek 4 wells chambered coverglasses (Thermo Scientific) were coated by adding 250 µl of 10 µg/ml FN in phosphate-buffered saline (PBS) and incubated for 1 hour at room temperature. Afterwards, the culture surface was removed of the FN solution and used for cell culture.

The polyacrylamide substrates with surface crosslinked FN were prepared following the procedure described by Tse and Engler [68]. In short, gels of approximately 7.4 kPa (or 35 kPa) stiffness and ∼100 µm thickness were prepared by mixing 10% acrylamide (BioRad) and 0.06% (or 0.3%) bisacrylamide (BioRad) with distilled water, adding 1% APS (Sigma) and 0.2% TEMED (Sigma), mixing, and sandwiching 60 µl of this solution between a glutaraldehyde (Sigma) activated coverslip (25 mm diameter) and a dichlorodimethylsilane (Sigma) passivated coverslip (15 mm diameter). After removal of the passivated coverslip, gels were rinsed with distilled water and activated by adding 100 µL NHS-diazirine (Sulfo-LC-SDA, 1 mg/ml in 50 mM HEPES pH 8.2, Pierce Biotechnology) and illuminating for 4 min under a strong 365 nm UV source. This step was repeated for a total of 3 times. Then, gels were quickly rinsed 2× with distilled water and 150 µl of a 50ug/ml solution of FN in 50 mM HEPES pH 8.2 were added and let react for 2 hours at room temperature. Gels were washed with PBS and sterilized by UV for 40 min in the cell culture hood.

### Cell spreading assays

For live imaging or ventral cell membrane isolation, 15000 trypsinized cells were plated in each well of the Lab-Tek 4 wells chambered coverglasses precoated with FN. Each sample well contained 500 µl culture media. Cell culture durations after cell seeding are indicated in the corresponding figure captions. For MLCK inhibition test, the trypsinized REF52-β3-integrin-EGFP cells were preincubated for 30 minutes using the culture media containing 30 or 40 µM of MLCK inhibitor ML-7 (Tocris), and then the cells were further cultured in the same treatment media during the experiment.

### Ventral cell membrane preparation

Fibroblasts cultured in the Lab-Tek 4 wells chambered coverglasses were extracellularly crosslinked with 250 µl solutions of *Bis*(NHS)PEO_5_ (Pierce) (0.1 mM in PBS, 20 minutes) at room temperature. This solution was then exchanged to 500 µl of nanopure water to incubate the sample for 10 minutes. Afterwards, the sample was rinsed with 250 µl of PBS three times and then stained for observation. This ventral cell membrane method well preserves the ventral cell membranes and their associated substrate adhesions and actin structures, as compared to the commonly used whole cell sample preparations, or other procedures for the ventral cell membrane preparation, such as lysis squirting [69]–[71] or sonication [72]. The removal of dorsal cell membrane excludes the overlapping signals from the dorsal membrane structures, thus enables an unequivocal identification of actual structural connections of the ventral actin structures with cell-substrate adhesions. In order to avoid the alternation to the ventral membrane structures, samples were not further fixed and were examined immediately after their preparation.

### Fluorescence staining

#### Cell membrane staining

Suspensions of the trypsinized cells were centrifuged (1200 rpm, 3 min, room temperature) and removed of the supernatant. The remaining cell pellet was resuspended in 1 ml of cell culture media containing the lipophilic membrane stain DiI (Invitrogen, 1∶250 dilution), which does not introduce noticeable changes to the cell behaviors. After incubation at 37°C for 10 min, the cell suspension was centrifuged (1200 rpm, 3 min, room temperature) and removed of the membrane dye containing supernatant. The remaining pellets of the membrane stained cells were resuspended in 1 ml of cell culture media and used for experiments.

#### Immunofluorescence staining

Lysed cells or ventral cell membrane samples in Lab-Tek 4 wells chambered coverglasses were removed of the original buffer solutions, gently rinsed three times with 250 µl of PBS, then added 250 µl solutions of the primary antibody (monoclonal mouse antibodies against human vinculin (Sigma), 1∶200 dilution in PBS) and incubated for 1 hour at room temperature. After the removal of the primary antibody solutions, the samples were gently rinsed three times with 250 µl of PBS, then added 250 µl solutions of the secondary antibodies (Cy5-conjugated donkey anti-mouse antibodies (Jackson Immuno), 1∶200 dilution in PBS) and incubated for 1 hour at room temperature. Afterwards, the secondary antibody solutions were removed and samples were gently rinsed three times with 250 µl of PBS.

#### Actin staining

Cell or ventral membrane samples were stained with 250 µl of Alex-488 (Invitrogen) or Fluoprobes-547 (FluoProbes; used in Fig. 7C) conjugated phalloidin1∶50 dilution in PBS) and incubated for 30 minutes at room temperature. Afterwards, the samples were removed of the staining solutions and rinsed three times with 250 µl of PBS. The stained samples were kept in PBS buffer for optical imaging.

### Optical microscopy

The confocal fluorescence, interference reflection, and differential interference contrast (DIC) imaging were carried out with an Olympus FV1000 confocal microscope (Olympus) and the FV10-ASW 1.7 software (Olympus). The images were collected with an oil-immersion UPlanSApo 1.35 NA 60× objective at room temperature, except that the time-lapse tracking of live cells was carried out at 37°C. Acquisition parameters including laser transmissivity, pixel dwell time, pinhole size, photomultiplier tube gains, and image size were adjusted for optimal detection sensitivity and minimal photobleaching.

#### Confocal fluorescence imaging

EGFP and Alex-488 were excited using the 488 nm laser line of an Ar ion laser. DiI was excited using the 543 laser line of a He-Ne laser. Cy5 was excited using the 633 nm laser line. The emissions of all fluorophores within 10 nm of bandwidth around their emission maxima were collected, except that the emissions of Cy5 was collected with BS 20/80 filter for the emissions greater than 640 nm. For multichannel imaging, the excitation and emission collection were carried out sequentially for each channel.

#### Interference reflection imaging

The 458 nm laser line of an Ar ion laser (1–2% of transmissivity) was used as incident light. The reflections at the bandwidth of 440–470 nm were collected. The imaging focus was set to the vicinity of the interface between cells or ventral membranes and the glass surface. This interface was found by changing the focus until the maximal reflection was obtained.

#### DIC imaging

Either 488 nm or 543 nm laser line was used as incident light and the transmitted light was collected.

#### Time-lapse imaging

The confocal microscope was equipped with a temperature and CO_2_ control chamber (Life Imaging Services, Switzerland) that maintained the entire microscope at 37°C, and saturated the sample with humidified air containing 5% CO_2_. The time-lapse imaging acquisition was automated with the FV10-ASW 1.7 software. Images were taken at the frame rate as indicated in figures and in captions of corresponding videos. Image series were converted into videos using ImageJ (Wayne Rasband, National Institutes of Health, http://rsb.info.nih.gov/ij/) and QuickTime Player 7 (Apple Inc.).

### Image Analysis

All images in 16 bit tiff format were processed with ImageJ or the FV10-ASW 1.6 Viewer (Olympus). When needed, images in original grey scale were contrast adjusted and applied false colours for presentation. Overlays of selected frames from the time-lapse sequences were performed in ImageJ. Overlays of images of different optical signals in the same field of view were performed with FV10-ASW 1.6 Viewer or ImageJ. Montages of the time-lapse sequences were made in ImageJ.

#### Kymographs

The kymograph is essentially the time-lapse montage of the cell edge region of defined width, which shows the trace of the cell edge position over time. The protrusion or retraction of the lamellipodium corresponds to the continuous increase or decrease of the cell edge positions respectively in the kymograph (Fig. S2C). Thus, the kinetic values of the protrusion or retraction of the lamellipodium, as well as of the net cell edge advancement, can be calculated accordingly. Kymographs were produced using ImageJ by taking 3-pixel-wide line in the time-lapse sequence. This 3-pixel-wide line was drawn perpendicular to the cell edge and to the direction of the cell edge advancement. The duration (t) and distance (d) in the movement of the lamellipodium were determined in the kymograph along the slopes as the x (time) and y (distance) values respectively, and the speed of movement was calculated accordingly as d/t (Fig. S2C). The quantification of the kinetics of lamellipodia used the kymographs based on DiI fluorescence or DIC signals. For the quantification of the net advancement speed of the cell edge, DIC, DiI fluorescence and diffusive intracellular EGFP background fluorescence were used alone or in combinations for an accurate identification of the cell edge position.

#### Quantifications of cell-substrate adhesions

For the cell-substrate adhesions identified as EGFP-β3-integrin clusters or vinculin immunostains, the regions of interest were cropped from the original images. The signal level of the background (non adhesion areas) in a cropped image was measured with the intensity profile along a line intersecting the adhesion. The original cropped images were then thresholded to only retain the adhesion signals. Alternatively for the images with fluorescence signals of the cell-substrate adhesions, the thresholding of the cropped images was done directly via the “make binary” routine in ImageJ. The adhesions in the threshold images were analyzed as particles (“analyze particles” routine in ImageJ) and we quantified their areas, widths, lengths and aspect ratios. The area was measured based on the total pixels of an adhesion. The length and width of an adhesion were determined respectively as the distances along the primary and secondary axis of the best fit ellipse. The aspect ratio was calculated as the length/width of the fitted ellipse.

#### Quantification of the fluorescence intensity of the cell-substrate adhesions

To quantify the fluorescence intensity, the β3-integrin-EGFP or vinculin clusters in the original image (in 8 bit format) were outlined by restoring the corresponding particle outlines in the thresholded image. After choosing “mean gray value” in the “set measurements” function, the “analyze particles” routine in ImageJ was used to measure the average fluorescence intensity, which is the mean grey value within the selection (the sum of the gray values of all the pixels in the selection divided by the number of pixels). The fluorescence intensity profile of the β3-integrin-EGFP cluster was generated using ImageJ with a 3-pixel-wide line drawn radially along the length of the filopodia adhesion in the last frame of its tracked sequence. The spatial start and end points of the measurement for the intensity profile correspond to the distal and proximal ends of the filopodia integrin cluster respectively.

#### Identification of filopodia adhesions in ventral cell membrane samples

They were determined as substrate adhesions in association with filopodia, or within ∼10 µm behind the filopodia containing cell edge (Fig. 7). Focal adhesions are characteristically associated with the radial oriented long dorsal stress fibers ([73] and Fig. S5C), and that the large focal adhesions in the early stage of spreading fibroblasts were located closer to the center of the cell (>10 µm from the cell edge).

### Statistics

Data analysis was carried out in Excel (Microsoft). Significance was determined using the t-test for the independent data groups of unequal variance. The P values (two tail) were given in the text. The P values less than 0.05 were considered significant.

## Supporting Information

Figure S1 **Growth kinetics of filopodia base adhesions. A.** Selected frames from the confocal microscopy time-lapse sequence of a growing β3-integrin-EGFP cluster associated with the filopodium base in a β3-integrin-EGFP (green) expressing REF52 fibroblast on FN coated glass (5 min–25 min after plating, Video S2). The cell edge and filopodium were visualized by the differential interference contrast (DIC) signal (grey). Formed behind the lamellipodium, the distal end of the filopodia base adhesion was in contact with the lamellipodium from the beginning of its formation. Along with the advancement of the lamellipodium, the filopodia base adhesion grew towards the filopodium tip. The dashed rectangle (top of the right cell image) was magnified in the left column (a–f). The white dashed vertical line is the reference line indicating the frontal elongation of the filopodia β3-integrin-EGFP cluster towards the filopodium tip by tightly following the advancing lamellipodium (a, b). This filopodia β3-integrin-EGFP cluster continuously widened (c, d, e, f) during its long duration of contact with the lamellipodium. Black arrows indicate the mobile non-adherent distal section of the filopodium, which was bended and recycled after the lamellipodium paused at the distal tip of the filopodia adhesion without net advancement. White arrows point to the newly formed β3-integrin-EGFP clusters extending circumferentially at the sides of the filopodia adhesion. White curves indicate the manually drawn contour of the lamellipodium based on the DIC contrast. The colored frames in A correspond to the colored phases as classified in B, i.e. the time period in which the β3-integrin-EGFP cluster contacts the advancing lamellipodium (yellow) and the events during pausing of the lamellipodium (pink) respectively. On the right column, the β3-integrin-EGFP images from different time points are overlaid (cyan-early, white-later). **B and C**. Quantified changes of size (B) and average fluorescence intensity (C) over time in the filopodia β3-integrin-EGFP cluster in A. The quantitative analyses were carried out and presented in the same way as in Fig. 1B for the filopodia tip adhesion. The fluorescence intensity of the integrin cluster increased (red arrows), and then it declined due to photobleaching (C). Black dashed vertical lines indicate the time points of the dynamic activities (bending, recycling) of the non-adherent distal sections of filopodia (FP). **D.** Quantitatively simialr occurrence (based on event counts) of the filopodia tip (black) and base (grey) adhesions in HFF (68.2%, 31.8%) and REF52-β3-integrin-EGFP (47.9%, 52.1%) cells spreading on FN coated glass. Scale bars: 5 µm (A, magnified views), 10 µm (A, overview).
(TIF)

Figure S2 **Kymographic characterization of the kinetics of cyclic protrusions and retractions of lamellipodia. A.** The time-lapse montage showing the cell edge dynamics (Fig. 3C, top) was represented by the kymographs. These kymographs (b) were generated with the fluorescence signal of the membrane lipid at sites indicated by the kymograph lines (3-pixel-wide, dashed yellow arrows in a). The dashed black arrows in a and b indicated the sequential positions of the kymograph lines (a) and the corresponding kymographs (b). Grey bars indicate the location of the filopodia adhesion. **B.** A HFF cell spreading on FN coated glass (51 min–64 min 20 s after plating, DIC, 10 s/frame). The white square region in a was magnified in b. The kymograph (c) was generated along the dashed yellow arrow in b, and showed the cyclic protrusions and retractions of the lamellipodium. **C.** Schematic cell edge trace as in kymograph, illustrating the quantification of the kinetic parameters of lamellipodium protrusions and retractions and the net advancement of the cell edge. **D.** Kinetic values of the periodic protrusions and retractions of lamellipodia in HFF and REF52-β3-integrin-EGFP cells spreading on FN coated glass (10 s/frame), in comparison to the previously reported kinetics of lamellipodia: (1) In lamellipodia associated with filopodia in neuronal growth cones [63]. (2) In isotropic spreading mouse embryonic fibroblast without filopodia [37]. *Values in parenthesis*: the number of measurements (at 9 filopodia adhesions in 3 REF52 cells, 5 sites with filopodia in 2 HFF cells). Scale bars: 5 µm (B-b), 10 µm (B-a).
(TIF)

Figure S3 **Quantification of the growth of the filopodia adhesions on PAA gel.** Time traces of the size growth (**A** and **B**) and the average fluorescence intensity (**C**) of the filopodia β3-integrin-EGFP cluster (presented in Fig. 4D–c, Video S6) in relation to the lamellipodium on soft (7.4 kPa) PAA gel. The analyses were performed and presented in the same way as for the filopodia adhesion in Fig. 1. **D.** The spatial distribution of β3-integrin in this filopodia adhesion. As described in Fig. 2E, the fluorescence intensity profile was generated along the long axis of the filopodia integrin cluster (insert: last frame of the tracking sequence, 3 µm×7 µm).
(TIF)

Figure S4 **Filopodia adhesions in HFF cells.** Spreading HFF cell (35–50 min (A), 15–25 min (B), 8–28 min (C), or 41–50 min (D) after plating on FN coated glass) were monitored by confocal interference reflection microscopy. **A and B.** Initiation and growth of tight substrate contacts from either the tip (A–b) or base (B–b) of filopodia. The cell edges (white rectangles in A–a and B–a) were shown in magnified views with the selected frames from the corresponding time-lapse sequences. Red arrows indicate the tight substrate contacts of filopodia. Brown arrows indicate the sections of filopodia in close proximity to the substrate surface. Yellow arrows indicate lamellipodia. Blue arrows indicate the non adherent distal section of filopodium bending at the distal tip of the filopodia adhesion. **C and D.** The fast advancing cell edges could either continue their fast advancement (C) or pause (D) after reaching the distal tips of filopodia adhesions (red arrows). **C.** Selected frames in the time-lapse sequence (Video S9) of the cell in fast spreading. **D.** Selected frames in the time-lapse sequence (Video S10) of the cell edge (dashed rectangles in D-a) showed that the cell edge advanced fast locally and then paused after reaching the distal tip of the filopodia adhesion. The movements of cell edges were represented by the kymographs (A–c, B–c, C–b, C–c) that were generated along the kymograph lines (dashed arrows) in the corresponding image sequences. Dashed lines drawn in the kymographs indicate the advancing (yellow) and pausing (pink) durations of lamellipodia at filopodia adhesions. The net advancement speeds of cell edges were measured at the corresponding sections of kymographs as indicated by dashed yellow lines (A–c, 9 nm/s; B–c, 58 nm/s; D–b, 100 nm/s; E–c, 92 nm/s). For comparison, the kymographs were horizontally stretched (A–c, 2.5 x; B–c, 1.5×; D–b, 3.5 x; E–c, 2.5 x) to have the same time scale. Scale bars: 2 µm (A–b, B–b), 5 µm (A–a, C, D–a,b), 10 µm (B–a).
(TIF)

Figure S5 **The growth kinetics of the adhesion formed at the lamellipodium type of cell edge (A and B). A.** Selected frames from the confocal microscopy time-lapse sequence of a growing β3-integrin-EGFP cluster in a β3-integrin-EGFP (green) expressing REF52 cell on FN coated glass (7 min–22 min after plating). The lamellipodium cell edge was identified by the DIC signal (grey). Formed in the lamellipodium, the adhesion (white arrows in b) grew into the cell lamellum. The dashed square in a was magnified in b at selected time points. **B.** Quantified growth of the adhesion initiated in the lamellipodium in its width (blue), length (red), area (black) and aspect ratio (grey). The same scales in vertical axes were used as for the filopodia adhesion in Fig. 1B for comparison. The growth characteristics of adhesions of lamellipodial origin were different from those of filopodia adhesions during the tracked early growth (<900 s). The length of the adhesion initiated at the lamellipodium type of cell edge was small and grew slowly without a stage of rapid increase, so that its aspect ratio only fluctuated slightly at very low values. **C.** The cytoskeletal organization at adhesions originated from lamellipodia. a. View of the exposed ventral surface of the cell edge region with no filopodia. This HFF cell (40 minutes plated on FN coated glass) partially retained its dorsal cell membrane structures (white asterisk). b,c,d. Magnified views in respective signals of the dashed rectangle region in a. The dorsal location of the remnant dorsal cell membrane structures (b and c, white asterisks) was confirmed by the higher membrane and actin fluorescences and the bright interference reflection signal in that region. The remnant dorsal cell membrane structures were tethered to the proximal sections of multiple radial oriented dorsal stress fibers. These dorsal stress fibers were anchored at their distal ends (d, pink arrows) in adhesions (c, pink arrows) of the size of focal complexes, which were originated from the lamellipodia type of cell edge. Scale bars: 1 µm (A–b), 2 µm (C–b), 5 µm (A–a, C–a).
(TIF)

Video S1 **The development of the filopodia tip adhesion in a spreading REF52-β3-integrin- EGFP cell.** (Fig. 1). Overlays of membrane lipid fluorescence (red) and β3-integrin-EGFP (green). The white rectangle region corresponds to the magnified view in Fig. 1A left column. 44 min 20 s–61 min 20 s after plating the cell on FN coated glass. The time-lapse seqeunce was collected at 20 s/frame with an Olympus FV1000 confocal microscope (Olympus). The video is shown at 5 frame/s.
(MOV)

Video S2 **The development of the filopodia base adhesion in a spreading REF52-β3-integrin- EGFP cell.** (Fig. S1). Overlays of DIC signal (grey) and β3-integrin-EGFP fluorescence (green). The white rectangle region corresponds to the magnified view in Fig. S1A left column. 5 min–25 min after plating the cell on FN coated glass. The time-lapse seqeunce was collected at 20 s/frame with an Olympus FV1000 confocal microscope. The video is shown at 5 frames/s.
(MOV)

Video S3 **The lamellipodium exhibited cyclic protrusions and retractions in the proximity of the growing filopodia adhesion in a spreading REF52-β3-integrin-EGFP cell.** (Fig. 3). Overlays of membrane lipid fluorescence (red) and β3-integrin-EGFP (green). The white rectangle region corresponds to the filopodia adhesion region analyzed in Fig. 3A–C. 20 min–47 min after plating the cell on FN coated glass. The time-lapse seqeunce was collected at 18 s/frame with an Olympus FV1000 confocal microscope. The video is shown at 5 frames/s.
(MOV)

Video S4 **The abolishment of the cyclic protrusions and retractions of the lamellipodium in a spreading REF52-β3-integrin- EGFP cell by MLCK inhibition with 30 µM of ML-7.** (Fig. 4A). Membrane lipid fluorescence signal (red). 25 min 3 s–29 min 58 s after plating the cell on FN coated glass. The white rectangle region corresponds to the cell edge region represented in montage. The time-lapse seqeunce was collected at 5 s/frame with an Olympus FV1000 confocal microscope. The video is shown at 5 frames/s.
(MOV)

Video S5 **Significant ruffling of the lamellipodium in a spreading REF52-β3-integrin-EGFP cell on soft PAA substrate due to the lack of effective surface adherence.** (Fig. 4C). Membrane lipid fluorescence signal (red). The white rectangle region corresponds to the cell edge region represented in montage. 103 min 14 s–113 min 14 s after plating the cell on FN coated PAA substrate. The time-lapse seqeunce was collected at 20 s/frame with an Olympus FV1000 confocal microscope. The video is shown at 5 frames/s.
(MOV)

Video S6 **The restored development of filopodia adhesion in the spreading REF52-β3-integrin-EGFP cell on soft PAA substrate with the locally suppressed ruffling of the lamellipodium.** (Fig. 4D). Overlays of membrane lipid fluorescence (red) and β3-integrin-EGFP (green). The white rectangle region corresponds to the filopodia adhesion region analyzed. 121 min 26 s–136 min 26 s after plating the cell on FN coated PAA substrate. The time-lapse seqeunce was collected at 30 s/frame with an Olympus FV1000 confocal microscope. The video is shown at 5 frames/s.
(MOV)

Video S7 **The rapidly disassembly of a filopodia adhesion when having very brief contact with the advancing lamellipodium in a spreading REF52-β3-integrin-EGFP cell.** (Fig. 5A). Overlays of membrane lipid fluorescence (red) and β3-integrin-EGFP (green). The white rectangle region corresponds to the filopodia adhesion region analyzed. 40 min 20 s–53 min 40 s after plating of the cell on FN coated glass. The time-lapse seqeunce was collected at 20 s/frame with an Olympus FV1000 confocal microscope. The video is shown at 5 frame/s. Frame size: 1.3 µm×4.4 µm.
(MOV)

Video S8 **During the fast spreading of a REF52-β3-integrin-EGFP cell, the fast advancing cell edge could rapidly surpass the filopodia adhesion and leave it behind in the cell lamellum, resulting in its rapid disassembly (at the horizontal arrow,** **Fig. 6B** **–b, I).** (Fig. 6B). Alternatively, the fast advancing cell edge could abruptly pause for long duration at the filopodia adhesion, resulting in its stable maturation (at the vertical arrow, Fig. 6B–b, II). Overlays of membrane lipid fluorescence (red) and β3-integrin-EGFP (green). 5 min–9 min 36 s after plating the cell on FN coated glass. The time-lapse seqeunce was collected at 12 s/frame with an Olympus FV1000 confocal microscope. The video is shown at 5 frames/s.
(MOV)

Video S9 **The rapid disassembly of filopodia adhesions due to their brief contact time with the fast advancing lamellipodium in a fast spreading HFF cell.** (Fig. S4C). Interference reflection signal. 8 min–28 min after plating the cell on FN coated glass. Red arrows: the dissembling filopodia adhesions. The time-lapse seqeunce was collected at 30 s/frame with an Olympus FV1000 confocal microscope. The video is shown at 5 frames/s.
(MOV)

Video S10 **The fast advancing cell edge paused at the distal tip of the filopodia adhesion in a spreading HFF cell.** (Fig. S4D). Interference reflection signal. The white square region corresponds to the filopodia adhesion region shown in Fig. S4D–b. Red arrow: the filopodia adhesion. 41 min–50 min after plating the cell on FN coated glass. The time-lapse seqeunce was collected at 20 s/frame with an Olympus FV1000 confocal microscope. The video is shown at 5 frame/s.
(MOV)

## References

[1] Galbraith CG, Yamada KM, Galbraith JA (2007) Polymerizing Actin Fibers Position Integrins Primed to Probe for Adhesion Sites. Science 315: 992–995. 17303755 10.1126/science.1137904

[2] Chan CE, Odde DJ (2008) Traction Dynamics of Filopodia on Compliant Substrates. Science 322: 1687–1691. 19074349 10.1126/science.1163595

[3] Kress H, Stelzer EHK, Holzer D, Buss F, Griffiths G, et al (2007) Filopodia act as phagocytic tentacles and pull with discrete steps and a load-dependent velocity. Proceedings of the National Academy of Sciences 104: 11633–11638. 10.1073/pnas.0702449104 PMC1913848 17620618

[4] Medalia O, Beck M, Ecke M, Weber I, Neujahr R, et al (2007) Organization of Actin Networks in Intact Filopodia. Current Biology 17: 79–84. 17208190 10.1016/j.cub.2006.11.022

[5] Bornschlögl T, Romero S, Vestergaard C, Joanny J, Van Nhieu G, et al (2013) Filopodial retraction force is generated by cortical actin dynamics and controlled by reversible tethering at the tip. Proc Natl Acad Sci U S A 110: 18928–18933. 24198333 10.1073/pnas.1316572110 PMC3839779

[6] Heckman CA, Plummer HK III (2013) Filopodia as sensors. Cellular Signalling 25: 2298–2311. 23876793 10.1016/j.cellsig.2013.07.006

[7] Cuvelier D, Théry M, Chu Y-S, Dufour S, Thiéry J-P, et al (2007) The Universal Dynamics of Cell Spreading. Current Biology 17: 694–699. 17379524 10.1016/j.cub.2007.02.058

[8] Albuschies J, Vogel V (2013) The role of filopodia in the recognition of nanotopographies. Sci Rep 3: 1658. 23584574 10.1038/srep01658 PMC3625890

[9] Choi C-H, Hagvall SH, Wu BM, Dunn JCY, Beygui RE, et al (2007) Cell interaction with three-dimensional sharp-tip nanotopography. Biomaterials 28: 1672–1679. 17174392 10.1016/j.biomaterials.2006.11.031

[10] Graves CE, McAllister RG, Rosoff WJ, Urbach JS (2009) Optical neuronal guidance in three-dimensional matrices. Journal of Neuroscience Methods 179: 278–283. 19428538 10.1016/j.jneumeth.2009.02.004 PMC3819723

[11] Cukierman E, Pankov R, Stevens DR, Yamada KM (2001) Taking Cell-Matrix Adhesions to the Third Dimension. Science 294: 1708–1712. 11721053 10.1126/science.1064829

[12] Gerhardt H, Golding M, Fruttiger M, Ruhrberg C, Lundkvist A, et al (2003) VEGF guides angiogenic sprouting utilizing endothelial tip cell filopodia. The Journal of Cell Biology 161: 1163–1177. 12810700 10.1083/jcb.200302047 PMC2172999

[13] Vignjevic D, Montagnac G (2008) Reorganisation of the dendritic actin network during cancer cell migration and invasion. Seminars in Cancer Biology 18: 12–22. 17928234 10.1016/j.semcancer.2007.08.001

[14] Dubin-Thaler BJ, Hofman JM, Cai Y, Xenias H, Spielman I, et al (2008) Quantification of Cell Edge Velocities and Traction Forces Reveals Distinct Motility Modules during Cell Spreading. PLoS ONE 3: e3735. 19011687 10.1371/journal.pone.0003735 PMC2581916

[15] Gupton SL, Gertler FB (2007) Filopodia: The Fingers That Do the Walking. Sci STKE 2007: re5. 17712139 10.1126/stke.4002007re5

[16] Mattila PK, Lappalainen P (2008) Filopodia: molecular architecture and cellular functions. Nat Rev Mol Cell Biol 9: 446–454. 18464790 10.1038/nrm2406

[17] DeRosier DJ, Edds KT (1980) Evidence for fascin cross-links between the actin filaments in coelomocyte filopodia. Experimental Cell Research 126: 490–494. 6892697 10.1016/0014-4827(80)90295-5

[18] Yang S, Huang F-K, Huang J, Chen S, Jakoncic J, et al (2013) Molecular Mechanism of Fascin Function in Filopodial Formation. Journal of Biological Chemistry 288: 274–284. 23184945 10.1074/jbc.M112.427971 PMC3537022

[19] Steketee MB, Tosney KW (2002) Three Functionally Distinct Adhesions in Filopodia: Shaft Adhesions Control Lamellar Extension. J Neurosci 22: 8071–8083. 12223561 10.1523/JNEUROSCI.22-18-08071.2002 PMC6758076

[20] Partridge MA, Marcantonio EE (2006) Initiation of Attachment and Generation of Mature Focal Adhesions by Integrin-containing Filopodia in Cell Spreading. Mol Biol Cell 17: 4237–4248. 16855018 10.1091/mbc.E06-06-0496 PMC1635363

[21] Nemethova M, Auinger S, Small JV (2008) Building the actin cytoskeleton: filopodia contribute to the construction of contractile bundles in the lamella. J Cell Biol 180: 1233–1244. 18362182 10.1083/jcb.200709134 PMC2290848

[22] Schäfer C, Borm B, Born S, Möhl C, Eibl E-M, et al (2009) One step ahead: Role of filopodia in adhesion formation during cell migration of keratinocytes. Experimental Cell Research 315: 1212–1224. 19100734 10.1016/j.yexcr.2008.11.008

[23] Möller J, Lühmann T, Chabria M, Hall H, Vogel V (2013) Macrophages lift off surface-bound bacteria using a filopodium-lamellipodium hook-and-shovel mechanism. Scientific Reports 3: 2884. 24097079 10.1038/srep02884 PMC3791455

[24] Cojoc D, Difato F, Ferrari E, Shahapure RB, Laishram J, et al (2007) Properties of the Force Exerted by Filopodia and Lamellipodia and the Involvement of Cytoskeletal Components. PLoS ONE 2: e1072. 17957254 10.1371/journal.pone.0001072 PMC2034605

[25] Oldenbourg R, Katoh K, Danuser G (2000) Mechanism of Lateral Movement of Filopodia and Radial Actin Bundles across Neuronal Growth Cones. Biophysical Journal 78: 1176–1182. 10692307 10.1016/S0006-3495(00)76675-6 PMC1300720

[26] Hytönen VP, Vogel V (2008) How Force Might Activate Talin's Vinculin Binding Sites: SMD Reveals a Structural Mechanism. PLoS Comput Biol 4: e24. 18282082 10.1371/journal.pcbi.0040024 PMC2242828

[27] del Rio A, Perez-Jimenez R, Liu R, Roca-Cusachs P, Fernandez JM, et al (2009) Stretching Single Talin Rod Molecules Activates Vinculin Binding. Science 323: 638–641. 19179532 10.1126/science.1162912 PMC9339221

[28] Zaidel-Bar R, Cohen M, Addadi L, Geiger B (2004) Hierarchical assembly of cell-matrix adhesion complexes. Biochem Soc Trans 32: 416–420. 15157150 10.1042/BST0320416

[29] Alexandrova AY, Arnold K, Schaub S, Vasiliev JM, Meister J-J, et al (2008) Comparative Dynamics of Retrograde Actin Flow and Focal Adhesions: Formation of Nascent Adhesions Triggers Transition from Fast to Slow Flow. PLoS ONE 3: e3234. 18800171 10.1371/journal.pone.0003234 PMC2535565

[30] Riveline D, Zamir E, Balaban NQ, Schwarz US, Ishizaki T, et al (2001) Focal Contacts as Mechanosensors: Externally Applied Local Mechanical Force Induces Growth of Focal Contacts by an mDia1-dependent and ROCK-independent Mechanism. J Cell Biol 153: 1175–1186. 11402062 10.1083/jcb.153.6.1175 PMC2192034

[31] Galbraith CG, Yamada KM, Sheetz MP (2002) The relationship between force and focal complex development. J Cell Biol 159: 695–705. 12446745 10.1083/jcb.200204153 PMC2173098

[32] Schäfer C, Born S, Möhl C, Houben S, Kirchgeßner N, et al (2010) The key feature for early migratory processes: Dependence of adhesion, actin bundles, force generation and transmission on filopodia. Cell Adhesion and Migration 4: 215–225. 20179423 10.4161/cam.4.2.10745 PMC2900617

[33] Roca-Cusachs P, del Rio A, Puklin-Faucher E, Gauthier NC, Biais N, et al.. (2013) Integrin-dependent force transmission to the extracellular matrix by α-actinin triggers adhesion maturation. Proceedings of the National Academy of Sciences. doi: 10.1073/pnas.1220723110. 10.1073/pnas.1220723110 PMC3625291 23515331

[34] Herbert BS, Michaela-Rosemarie H, Julien P, Timothée V, Sara Z, et al (2013) β1- and αv-class integrins cooperate to regulate myosin II during rigidity sensing of fibronectin-based microenvironments. Nature cell biology 15: 625–636. 23708002 10.1038/ncb2747

[35] Giannone G, Dubin-Thaler BJ, Rossier O, Cai Y, Chaga O, et al (2007) Lamellipodial Actin Mechanically Links Myosin Activity with Adhesion-Site Formation. Cell 128: 561–575. 17289574 10.1016/j.cell.2006.12.039 PMC5219974

[36] Choi CK, Vicente-Manzanares M, Zareno J, Whitmore LA, Mogilner A, et al (2008) Actin and [alpha]-actinin orchestrate the assembly and maturation of nascent adhesions in a myosin II motor-independent manner. Nat Cell Biol 10: 1039–1050. 19160484 10.1038/ncb1763 PMC2827253

[37] Giannone G, Dubin-Thaler BJ, Döbereiner H-G, Kieffer N, Bresnick AR, et al (2004) Periodic Lamellipodial Contractions Correlate with Rearward Actin Waves. Cell 116: 431–443. 15016377 10.1016/s0092-8674(04)00058-3

[38] Pollard TD, Borisy GG (2003) Cellular Motility driven by assembly and disassembly of actin filaments. Cell 112: 453–465. 12600310 10.1016/s0092-8674(03)00120-x

[39] Verkhovsky AB, Svitikina TM, Borisy GG (1999) Network contraction model for cell translocation and retrograde flow. Biochem Soc Symp 65: 207–222. 10320940

[40] Hall A (1998) Rho GTPases and the Actin Cytoskeleton. Science 279: 509–514. 9438836 10.1126/science.279.5350.509

[41] Aspenström P, Fransson A, Saras J (2004) Rho GTPases have diverse effects on the organization of the actin filament system. Biochem J 377: 327–337. 14521508 10.1042/BJ20031041 PMC1223866

[42] Mejillano MR, Kojima S-i, Applewhite DA, Gertler FB, Svitkina TM, et al (2004) Lamellipodial Versus Filopodial Mode of the Actin Nanomachinery: Pivotal Role of the Filament Barbed End. Cell 118: 363–373. 15294161 10.1016/j.cell.2004.07.019

[43] Vignjevic D, Kojima S-i, Aratyn Y, Danciu O, Svitkina T, et al (2006) Role of fascin in filopodial protrusion. J Cell Biol 174: 863–875. 16966425 10.1083/jcb.200603013 PMC2064340

[44] Insall RH, Machesky LM (2009) Actin Dynamics at the Leading Edge: From Simple Machinery to Complex Networks. 17: 310–322. 10.1016/j.devcel.2009.08.012 19758556

[45] Ballestrem C, Hinz B, Imhof BA, Wehrle-Haller B (2001) Marching at the front and dragging behind: differential αVβ3-integrin turnover regulates focal adhesion behavior. J Cell Biol 155: 1319–1332. 11756480 10.1083/jcb.200107107 PMC2199321

[46] Roca-Cusachs P, Gauthier NC, del Rio A, Sheetz MP (2009) Clustering of α5β1 integrins determines adhesion strength whereas αvβ3 and talin enable mechanotransduction. Proceedings of the National Academy of Sciences 106: 16245–16250. 10.1073/pnas.0902818106 PMC2752568 19805288

[47] Jiang G, Huang AH, Cai Y, Tanase M, Sheetz MP (2006) Rigidity Sensing at the Leading Edge through [alpha]v[beta]3 Integrins and RPTP[alpha]. Biophysical Journal 90: 1804–1809. 16339875 10.1529/biophysj.105.072462 PMC1367329

[48] Cluzel C, Saltel F, Lussi J, Paulhe F, Imhof BA, et al (2005) The mechanisms and dynamics of {alpha}v{beta}3 integrin clustering in living cells. J Cell Biol 171: 383–392. 16247034 10.1083/jcb.200503017 PMC2171205

[49] Hinz B, Dugina V, Ballestrem C, Wehrle-Haller B, Chaponnier C (2003) {alpha}-Smooth Muscle Actin Is Crucial for Focal Adhesion Maturation in Myofibroblasts. Mol Biol Cell 14: 2508–2519. 12808047 10.1091/mbc.E02-11-0729 PMC194898

[50] Saltel F, Mortier E, Hytönen VP, Jacquier M-C, Zimmermann P, et al (2009) New PI(4,5)P2- and membrane proximal integrin–binding motifs in the talin head control β3-integrin clustering. The Journal of Cell Biology 187: 715–731. 19948488 10.1083/jcb.200908134 PMC2806581

[51] Grashoff C, Hoffman BD, Brenner MD, Zhou R, Parsons M, et al (2010) Measuring mechanical tension across vinculin reveals regulation of focal adhesion dynamics. Nature 466: 263–266. 20613844 10.1038/nature09198 PMC2901888

[52] Yu C-h, Law JBK, Suryana M, Low HY, Sheetz MP (2011) Early integrin binding to Arg-Gly-Asp peptide activates actin polymerization and contractile movement that stimulates outward translocation. Proceedings of the National Academy of Sciences. doi: 10.1073/pnas.1109485108. 10.1073/pnas.1109485108 PMC3251131 22139375

[53] Ji L, Lim J, Danuser G (2008) Fluctuations of intracellular forces during cell protrusion. Nat Cell Biol 10: 1393–1400. 19011623 10.1038/ncb1797 PMC2597050

[54] Balaban NQ, Schwarz US, Riveline D, Goichberg P, Tzur G, et al (2001) Force and focal adhesion assembly: a close relationship studied using elastic micropatterned substrates. Nat Cell Biol 3: 466–472. 11331874 10.1038/35074532

[55] Koestler SA, Auinger S, Vinzenz M, Rottner K, Small JV (2008) Differentially oriented populations of actin filaments generated in lamellipodia collaborate in pushing and pausing at the cell front. Nat Cell Biol 10: 306–313. 18278037 10.1038/ncb1692

[56] Verkhovsky AB, Svitkina TM, Borisy GG (1995) Myosin II filament assemblies in the active lamella of fibroblasts: their morphogenesis and role in the formation of actin filament bundles. J Cell Biol 131: 989–1002. 7490299 10.1083/jcb.131.4.989 PMC2200006

[57] Pelham RJ, Wang Y-l (1997) Cell locomotion and focal adhesions are regulated by substrate flexibility. Proceedings of the National Academy of Sciences 94: 13661–13665. 10.1073/pnas.94.25.13661 PMC28362 9391082

[58] Nobes CD, Hall A (1995) Rho, Rac, and Cdc42 GTPases regulate the assembly of multimolecular focal complexes associated with actin stress fibers, lamellipodia, and filopodia. Cell 81: 53–62. 7536630 10.1016/0092-8674(95)90370-4

[59] Shahapure R, Difato F, Laio A, Bisson G, Ercolini E, et al (2010) Force Generation in Lamellipodia Is a Probabilistic Process with Fast Growth and Retraction Events. Biophysical Journal 98: 979–988. 20303855 10.1016/j.bpj.2009.11.041 PMC2849058

[60] Ponti A, Machacek M, Gupton SL, Waterman-Storer CM, Danuser G (2004) Two Distinct Actin Networks Drive the Protrusion of Migrating Cells. Science 305: 1782–1786. 15375270 10.1126/science.1100533

[61] Gardel ML, Sabass B, Ji L, Danuser G, Schwarz US, et al (2008) Traction stress in focal adhesions correlates biphasically with actin retrograde flow speed. J Cell Biol 183: 999–1005. 19075110 10.1083/jcb.200810060 PMC2600750

[62] Shemesh T, Verkhovsky AB, Svitkina TM, Bershadsky AD, Kozlov MM (2009) Role of Focal Adhesions and Mechanical Stresses in the Formation and Progression of the Lamellum Interface. Biophysical Journal 97: 1254–1264. 19720013 10.1016/j.bpj.2009.05.065 PMC2749772

[63] Mongiu AK, Weitzke EL, Chaga OY, Borisy GG (2007) Kinetic-structural analysis of neuronal growth cone veil motility. Journal of Cell Science 120: 1113–1125. 17327278 10.1242/jcs.03384

[64] Burnette DT, Manley S, Sengupta P, Sougrat R, Davidson MW, et al (2011) A role for actin arcs in the leading-edge advance of migrating cells. Nat Cell Biol 13: 371–382. 21423177 10.1038/ncb2205 PMC3646481

[65] Lowery LA, Vactor DV (2009) The trip of the tip: understanding the growth cone machinery. Nat Rev Mol Cell Biol 10: 332–343. 19373241 10.1038/nrm2679 PMC2714171

[66] Gallo G (2011) The Neuronal Actin Cytoskeleton and the Protrusion of Lamellipodia and Filopodia. In: Gallo G, Lanier LM, editors. Neurobiology of Actin. New York: Springer. 7–22.

[67] Engvall E, Ruoslahti E (1977) Binding of soluble form of fibroblast surface protein, fibronectin, to collagen. International Journal of Cancer 20: 1–5. 903179 10.1002/ijc.2910200102

[68] Tse JR, Engler AJ (2001) Preparation of Hydrogel Substrates with Tunable Mechanical Properties. Current Protocols in Cell Biology. Hoboken, NJ: John Wiley & Sons, Inc. 10.1002/0471143030.cb1016s47 20521229

[69] Avnur Z, Geiger B (1981) Substrate-attached membranes of cultured cells isolation and characterization of ventral cell membranes and the associated cytoskeleton. Journal of Molecular Biology 153: 361–379. 7040683 10.1016/0022-2836(81)90283-7

[70] Lang R, Nermut M, Williams L (1981) Ultrastructure of sheep erythrocyte plasma membranes and cytoskeletons bound to solid supports. J Cell Sci 49: 383–399. 7309811 10.1242/jcs.49.1.383

[71] Nermut MV (1995) Manipulation of Cell Monolayers to Reveal Plasma Membrane Surfaces for Freeze-Drying and Surface Replication. In: Severs NJ, Shotton DM, editors. Rapid Freezing, Freeze Fracture and Deep Etching. Wilmington, DE: Wiley-Liss, Inc. 151–172.

[72] Yamada S, Pokutta S, Drees F, Weis WI, Nelson WJ (2005) Deconstructing the Cadherin-Catenin-Actin Complex. 123: 889–901. 10.1016/j.cell.2005.09.020 PMC3368712 16325582

[73] Hotulainen P, Lappalainen P (2006) Stress fibers are generated by two distinct actin assembly mechanisms in motile cells. J Cell Biol 173: 383–394. 16651381 10.1083/jcb.200511093 PMC2063839

