# Supplementary figures and images for: Maturation of Filopodia Shaft Adhesions Is Upregulated by Local Cycles of Lamellipodia Advancements and Retractions
Source: PLoS One. 2014 Sep 17;9(9):e107097. doi: 10.1371/journal.pone.0107097 (PMC4167701; doi:10.1371/journal.pone.0107097)

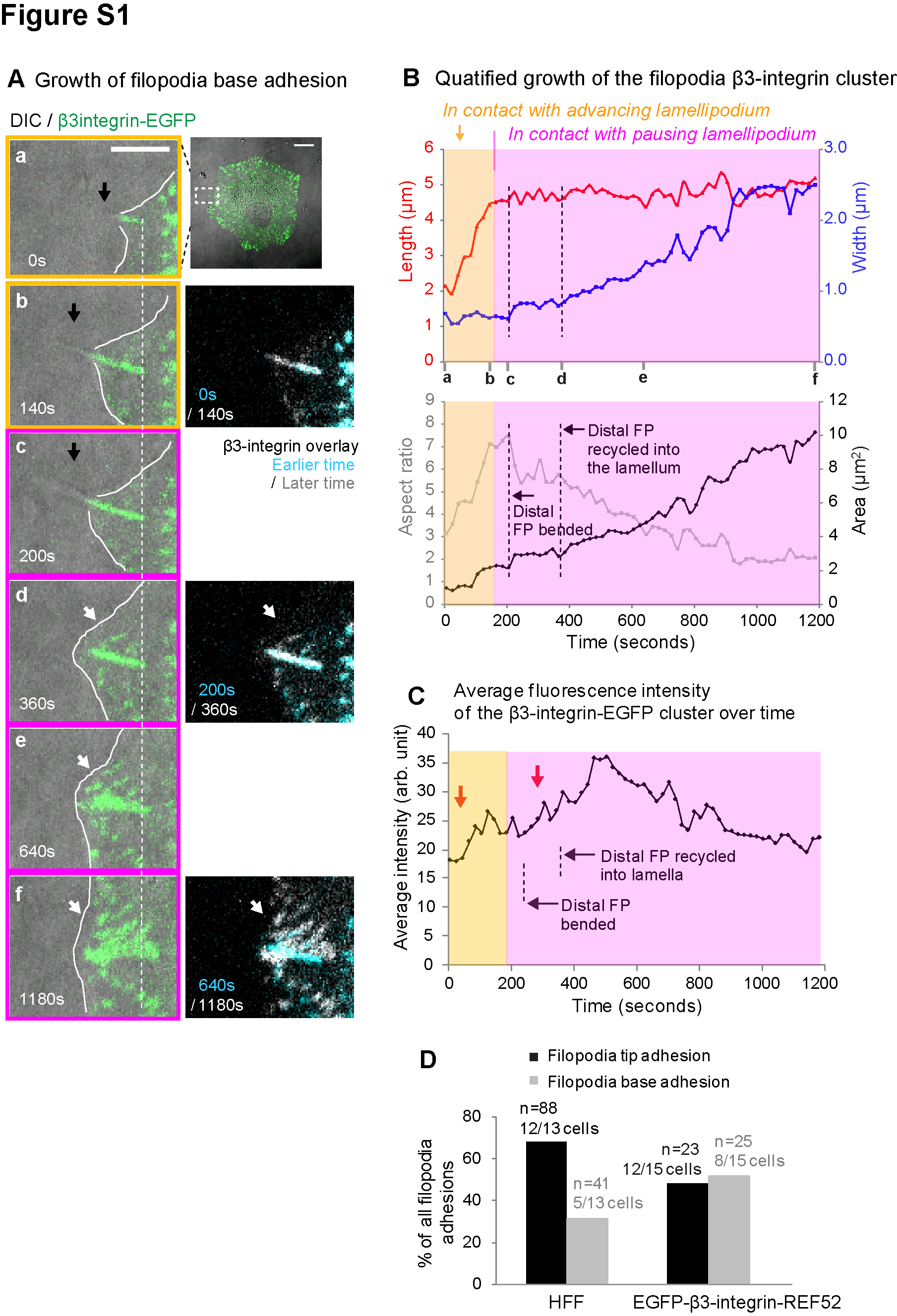

Supplement: Figure S1 — Growth kinetics of filopodia base adhesions. A. Selected frames from the confocal microscopy time-lapse sequence of a growing β3-integrin-EGFP cluster associated with the filopodium base in a β3-integrin-EGFP (green) expressing REF52 fibroblast on FN coated glass (5 min–25 min after plating, Video S2). The cell edge and filopodium were visualized by the differential interference contrast (DIC) signal (grey). Formed behind the lamellipodium, the distal end of the filopodia base adhesion was in contact with the lamellipodium from the beginning of its formation. Along with the advancement of the lamellipodium, the filopodia base adhesion grew towards the filopodium tip. The dashed rectangle (top of the right cell image) was magnified in the left column (a–f). The white dashed vertical line is the reference line indicating the frontal elongation of the filopodia β3-integrin-EGFP cluster towards the filopodium tip by tightly following the advancing lamellipodium (a, b). This filopodia β3-integrin-EGFP cluster continuously widened (c, d, e, f) during its long duration of contact with the lamellipodium. Black arrows indicate the mobile non-adherent distal section of the filopodium, which was bended and recycled after the lamellipodium paused at the distal tip of the filopodia adhesion without net advancement. White arrows point to the newly formed β3-integrin-EGFP clusters extending circumferentially at the sides of the filopodia adhesion. White curves indicate the manually drawn contour of the lamellipodium based on the DIC contrast. The colored frames in A correspond to the colored phases as classified in B, i.e. the time period in which the β3-integrin-EGFP cluster contacts the advancing lamellipodium (yellow) and the events during pausing of the lamellipodium (pink) respectively. On the right column, the β3-integrin-EGFP images from different time points are overlaid (cyan-early, white-later). B and C. Quantified changes of size (B) and average fluorescence intensity ( [file pone.0107097.s001.tif]

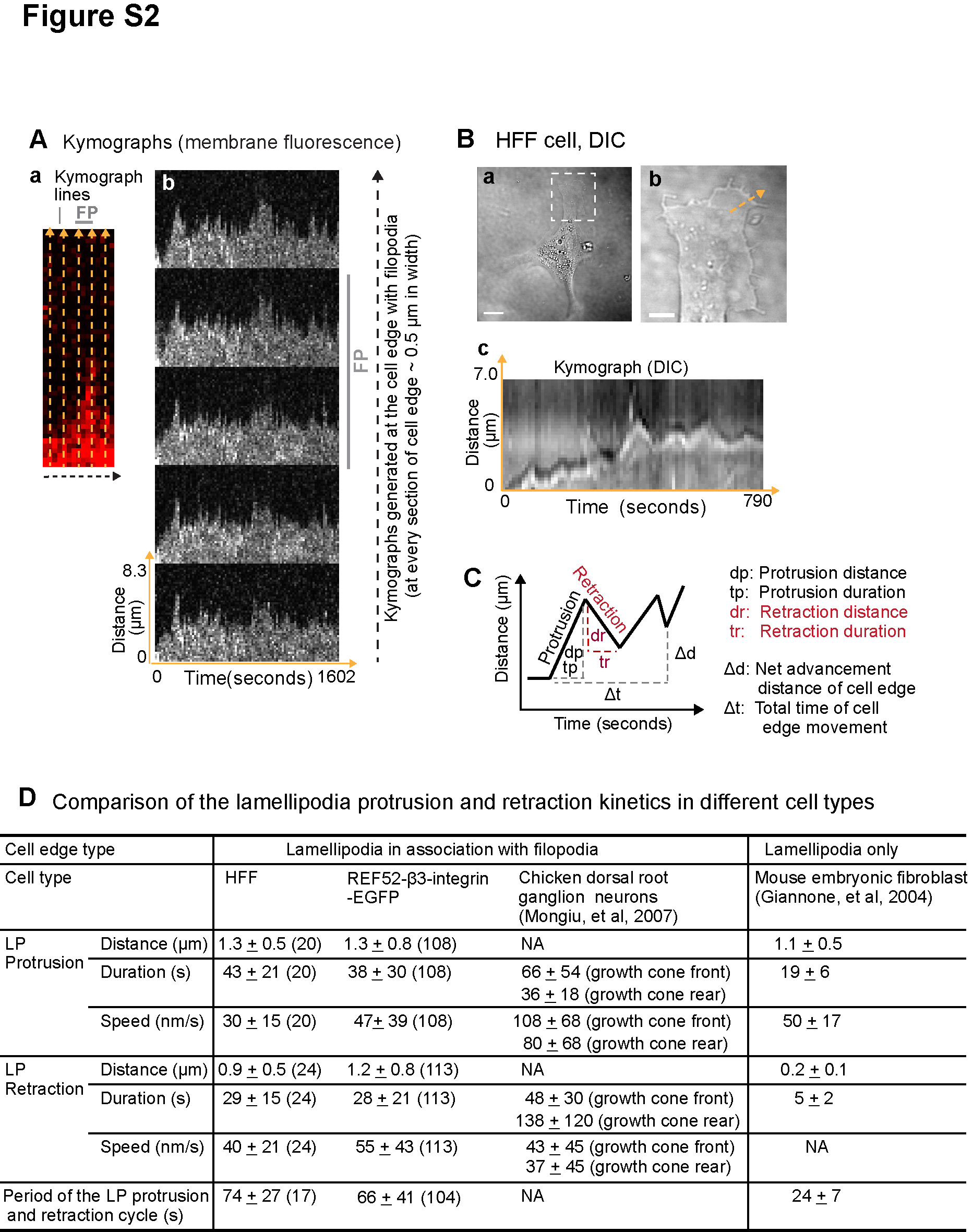

Supplement: Figure S2 — Kymographic characterization of the kinetics of cyclic protrusions and retractions of lamellipodia. A. The time-lapse montage showing the cell edge dynamics (Fig. 3C, top) was represented by the kymographs. These kymographs (b) were generated with the fluorescence signal of the membrane lipid at sites indicated by the kymograph lines (3-pixel-wide, dashed yellow arrows in a). The dashed black arrows in a and b indicated the sequential positions of the kymograph lines (a) and the corresponding kymographs (b). Grey bars indicate the location of the filopodia adhesion. B. A HFF cell spreading on FN coated glass (51 min–64 min 20 s after plating, DIC, 10 s/frame). The white square region in a was magnified in b. The kymograph (c) was generated along the dashed yellow arrow in b, and showed the cyclic protrusions and retractions of the lamellipodium. C. Schematic cell edge trace as in kymograph, illustrating the quantification of the kinetic parameters of lamellipodium protrusions and retractions and the net advancement of the cell edge. D. Kinetic values of the periodic protrusions and retractions of lamellipodia in HFF and REF52-β3-integrin-EGFP cells spreading on FN coated glass (10 s/frame), in comparison to the previously reported kinetics of lamellipodia: (1) In lamellipodia associated with filopodia in neuronal growth cones [63]. (2) In isotropic spreading mouse embryonic fibroblast without filopodia [37]. Values in parenthesis: the number of measurements (at 9 filopodia adhesions in 3 REF52 cells, 5 sites with filopodia in 2 HFF cells). Scale bars: 5 µm (B-b), 10 µm (B-a). (TIF) [file pone.0107097.s002.tif]

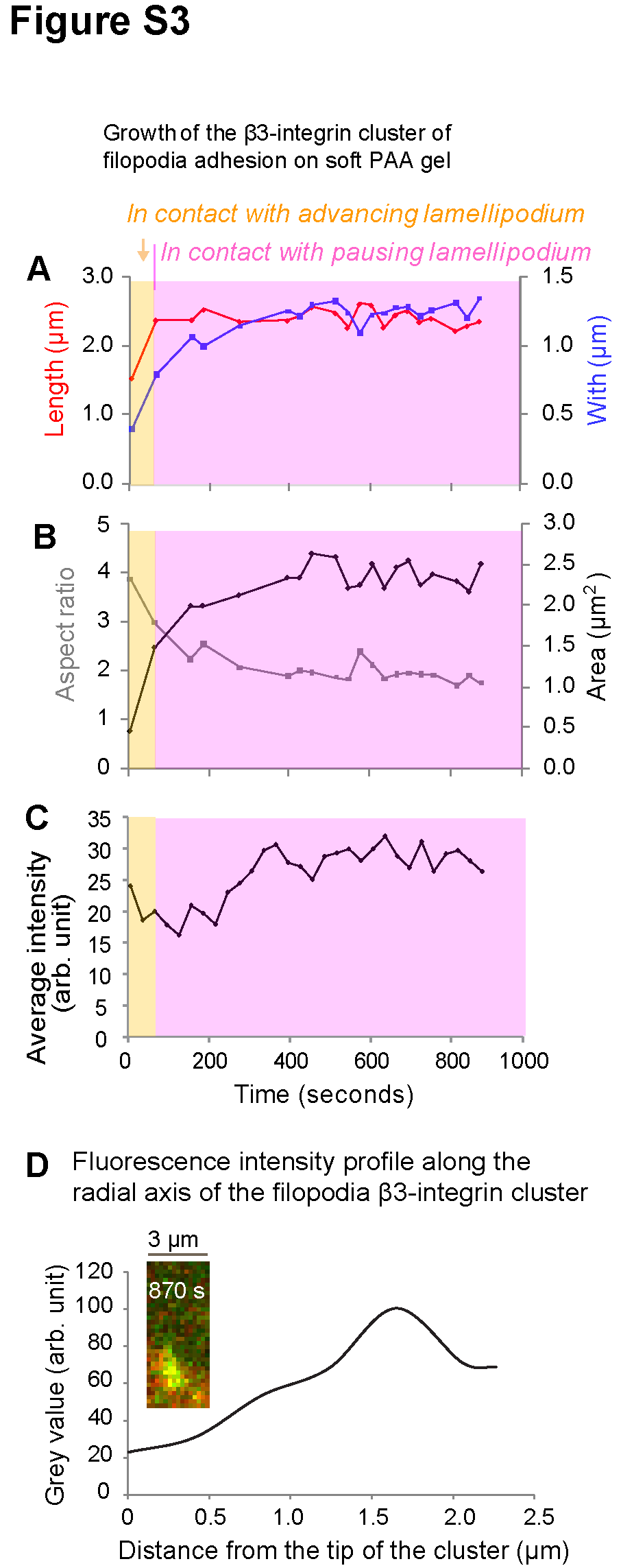

Supplement: Figure S3 — Quantification of the growth of the filopodia adhesions on PAA gel. Time traces of the size growth (A and B) and the average fluorescence intensity (C) of the filopodia β3-integrin-EGFP cluster (presented in Fig. 4D–c, Video S6) in relation to the lamellipodium on soft (7.4 kPa) PAA gel. The analyses were performed and presented in the same way as for the filopodia adhesion in Fig. 1. D. The spatial distribution of β3-integrin in this filopodia adhesion. As described in Fig. 2E, the fluorescence intensity profile was generated along the long axis of the filopodia integrin cluster (insert: last frame of the tracking sequence, 3 µm×7 µm). (TIF) [file pone.0107097.s003.tif]

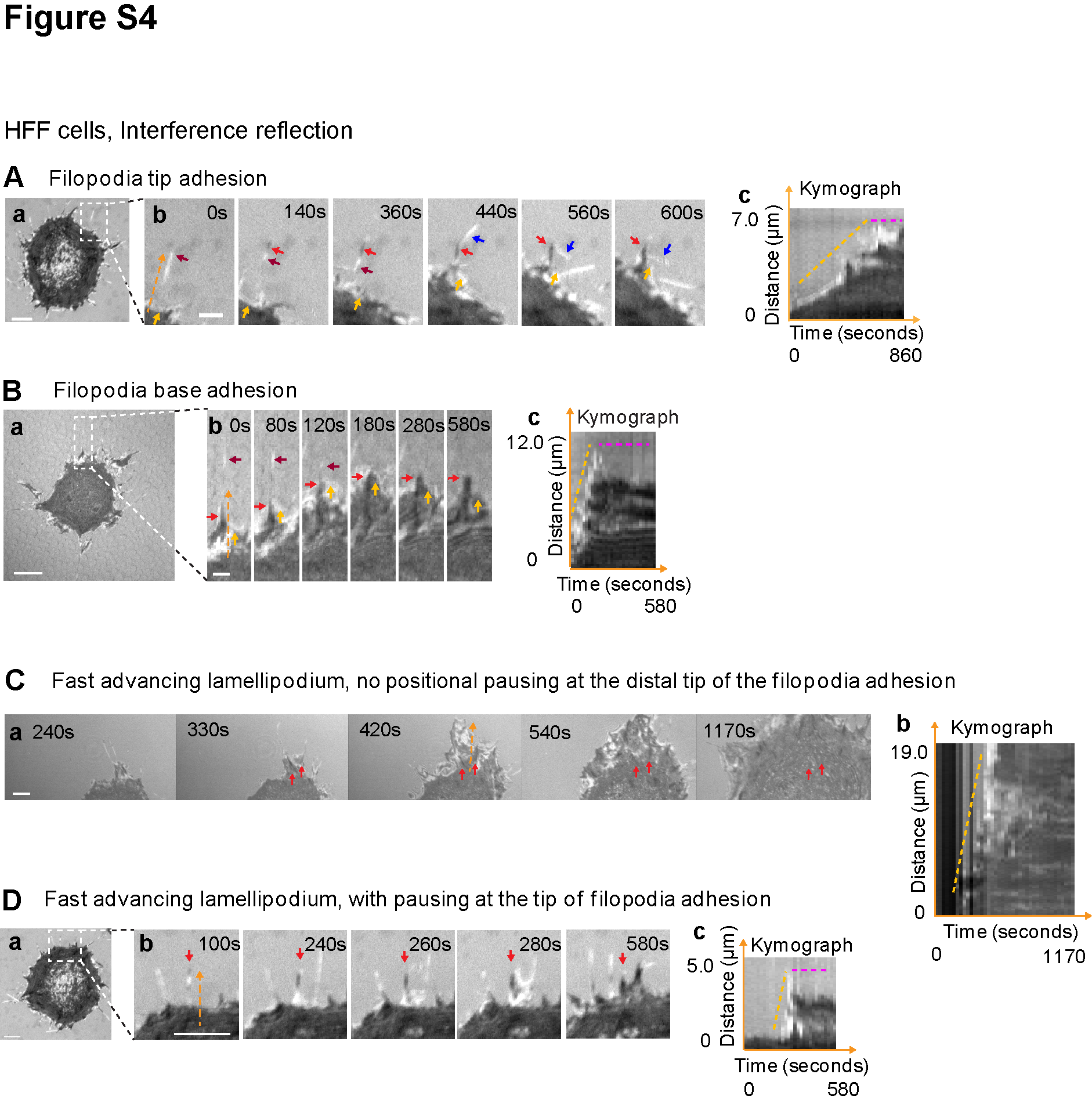

Supplement: Figure S4 — Filopodia adhesions in HFF cells. Spreading HFF cell (35–50 min (A), 15–25 min (B), 8–28 min (C), or 41–50 min (D) after plating on FN coated glass) were monitored by confocal interference reflection microscopy. A and B. Initiation and growth of tight substrate contacts from either the tip (A–b) or base (B–b) of filopodia. The cell edges (white rectangles in A–a and B–a) were shown in magnified views with the selected frames from the corresponding time-lapse sequences. Red arrows indicate the tight substrate contacts of filopodia. Brown arrows indicate the sections of filopodia in close proximity to the substrate surface. Yellow arrows indicate lamellipodia. Blue arrows indicate the non adherent distal section of filopodium bending at the distal tip of the filopodia adhesion. C and D. The fast advancing cell edges could either continue their fast advancement (C) or pause (D) after reaching the distal tips of filopodia adhesions (red arrows). C. Selected frames in the time-lapse sequence (Video S9) of the cell in fast spreading. D. Selected frames in the time-lapse sequence (Video S10) of the cell edge (dashed rectangles in D-a) showed that the cell edge advanced fast locally and then paused after reaching the distal tip of the filopodia adhesion. The movements of cell edges were represented by the kymographs (A–c, B–c, C–b, C–c) that were generated along the kymograph lines (dashed arrows) in the corresponding image sequences. Dashed lines drawn in the kymographs indicate the advancing (yellow) and pausing (pink) durations of lamellipodia at filopodia adhesions. The net advancement speeds of cell edges were measured at the corresponding sections of kymographs as indicated by dashed yellow lines (A–c, 9 nm/s; B–c, 58 nm/s; D–b, 100 nm/s; E–c, 92 nm/s). For comparison, the kymographs were horizontally stretched (A–c, 2.5 x; B–c, 1.5×; D–b, 3.5 x; E–c, 2.5 x) to have the same time scale. Scale bars: 2 µm (A–b, B–b), 5 µm (A–a, C, D–a,b), 10 µm (B–a). (TIF) [file pone.0107097.s004.tif]

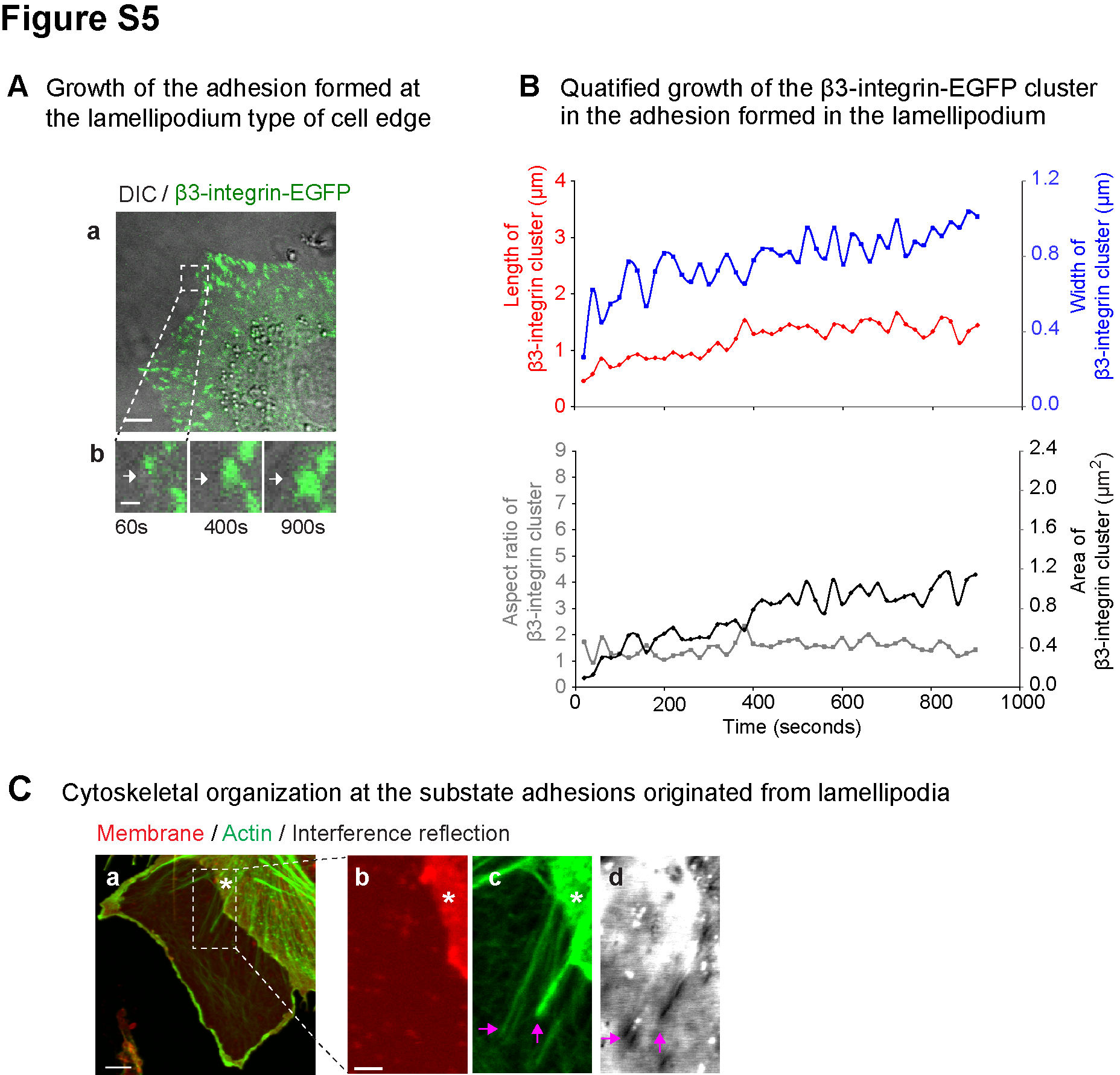

Supplement: Figure S5 — The growth kinetics of the adhesion formed at the lamellipodium type of cell edge (A and B). A. Selected frames from the confocal microscopy time-lapse sequence of a growing β3-integrin-EGFP cluster in a β3-integrin-EGFP (green) expressing REF52 cell on FN coated glass (7 min–22 min after plating). The lamellipodium cell edge was identified by the DIC signal (grey). Formed in the lamellipodium, the adhesion (white arrows in b) grew into the cell lamellum. The dashed square in a was magnified in b at selected time points. B. Quantified growth of the adhesion initiated in the lamellipodium in its width (blue), length (red), area (black) and aspect ratio (grey). The same scales in vertical axes were used as for the filopodia adhesion in Fig. 1B for comparison. The growth characteristics of adhesions of lamellipodial origin were different from those of filopodia adhesions during the tracked early growth (<900 s). The length of the adhesion initiated at the lamellipodium type of cell edge was small and grew slowly without a stage of rapid increase, so that its aspect ratio only fluctuated slightly at very low values. C. The cytoskeletal organization at adhesions originated from lamellipodia. a. View of the exposed ventral surface of the cell edge region with no filopodia. This HFF cell (40 minutes plated on FN coated glass) partially retained its dorsal cell membrane structures (white asterisk). b,c,d. Magnified views in respective signals of the dashed rectangle region in a. The dorsal location of the remnant dorsal cell membrane structures (b and c, white asterisks) was confirmed by the higher membrane and actin fluorescences and the bright interference reflection signal in that region. The remnant dorsal cell membrane structures were tethered to the proximal sections of multiple radial oriented dorsal stress fibers. These dorsal stress fibers were anchored at their distal ends (d, pink arrows) in adhesions (c, pink arrows) of the size of focal complexes, which were or [file pone.0107097.s005.tif]
